# Identifying Immunological and Clinical Predictors of COVID-19 Severity and Sequelae by Mathematical Modeling

**DOI:** 10.3389/fimmu.2022.865845

**Published:** 2022-04-20

**Authors:** Noha M. Elemam, Sarah Hammoudeh, Laila Salameh, Bassam Mahboub, Habiba Alsafar, Iman M. Talaat, Peter Habib, Mehmood Siddiqui, Khalid Omar Hassan, Omar Yousef Al-Assaf, Jalal Taneera, Nabil Sulaiman, Rifat Hamoudi, Azzam A. Maghazachi, Qutayba Hamid, Maha Saber-Ayad

**Affiliations:** ^1^ College of Medicine, University of Sharjah, Sharjah, United Arab Emirates; ^2^ Sharjah Institute for Medical Research, University of Sharjah, Sharjah, United Arab Emirates; ^3^ Dubai Health Authority, Rashid Hospital, Dubai, United Arab Emirates; ^4^ Center for Biotechnology, Khalifa University of Science and Technology, Abu Dhabi, United Arab Emirates; ^5^ Department of Biomedical Engineering, College of Engineering, Khalifa University of Science and Technology, Abu Dhabi, United Arab Emirates; ^6^ Department of Genetics and Molecular Biology, College of Medicine and Health Sciences, Khalifa University of Science and Technology, Abu Dhabi, United Arab Emirates; ^7^ Emirates Bio-Research Centre, Ministry of Interior, Abu Dhabi, United Arab Emirates; ^8^ Pathology Department, Faculty of Medicine, Alexandria University, Alexandria, Egypt; ^9^ School of Information Technology and Computer Science (ITCS), Nile University, Giza, Egypt; ^10^ Division of Surgery and Interventional Science, University College London, London, United Kingdom; ^11^ Meakins-Christie Laboratories, Research Institute of the McGill University Health Center, Montreal, QC, Canada; ^12^ College of Medicine, Cairo University, Giza, Egypt

**Keywords:** COVID-19, RNA seq, transcriptomics, multiplex, ROC analysis, Aritficial Intelligence, Machine Learning

## Abstract

Since its emergence as a pandemic in March 2020, coronavirus disease (COVID-19) outcome has been explored *via* several predictive models, using specific clinical or biochemical parameters. In the current study, we developed an integrative non-linear predictive model of COVID-19 outcome, using clinical, biochemical, immunological, and radiological data of patients with different disease severities. Initially, the immunological signature of the disease was investigated through transcriptomics analysis of nasopharyngeal swab samples of patients with different COVID-19 severity versus control subjects (exploratory cohort, n=61), identifying significant differential expression of several cytokines. Accordingly, 24 cytokines were validated using a multiplex assay in the serum of COVID-19 patients and control subjects (validation cohort, n=77). Predictors of severity were Interleukin (IL)-10, Programmed Death-Ligand-1 (PDL-1), Tumor necrosis factors-α, absolute neutrophil count, C-reactive protein, lactate dehydrogenase, blood urea nitrogen, and ferritin; with high predictive efficacy (AUC=0.93 and 0.98 using ROC analysis of the predictive capacity of cytokines and biochemical markers, respectively). Increased IL-6 and granzyme B were found to predict liver injury in COVID-19 patients, whereas interferon-gamma (IFN-γ), IL-1 receptor-a (IL-1Ra) and PD-L1 were predictors of remarkable radiological findings. The model revealed consistent elevation of IL-15 and IL-10 in severe cases. Combining basic biochemical and radiological investigations with a limited number of curated cytokines will likely attain accurate predictive value in COVID-19. The model-derived cytokines highlight critical pathways in the pathophysiology of the COVID-19 with insight towards potential therapeutic targets. Our modeling methodology can be implemented using new datasets to identify key players and predict outcomes in new variants of COVID-19.

## 1 Introduction

The coronavirus disease (COVID-19) has been following a non-linear evolution through the pandemic, starting with one variant that mutated into at least four dominant subtypes. Early prediction of COVID-19 outcome is crucial to direct resource allocation by the health care system and to triage the patients to receive the optimum clinical management. Despite the broad spectrum of presentations, a significant turning point in the course of the disease is the development of abrupt systemic elevation of a myriad of inflammatory cytokines and chemokines (the cytokine storm- CS). In this phase of COVID-19, multiple organ failure progressing to circulatory shock is the leading cause of death. The CS is accompanied by a myriad of biochemical and radiological findings (1). A key determinant factor of COVID19 progression is the uncontrolled dysregulation production of cytokines and chemokines, resulting in the development of a cytokine storm, systemic inflammation, and consequently multi-organ failure (2). The presence of the cytokine storm was associated with COVID-19 severity as previously reported (3, 4), where the serum levels of cytokines in COVID-19 patients were significantly correlated with the severity of the disease and acted as warning indicators of the severity and progression of COVID-19.

Interpreting the role of cytokines, their predictive value and therapeutic potential is still a significant challenge in the context of COVID-19. An example of an incomplete understanding of CS and its pathogenesis is the uprise and drop of tocilizumab. As interleukin-6 (IL-6) is a critical cytokine in CS-induced mortality in patients receiving engineered T cell therapy, it was first suggested as a potential therapeutic target for COVID-19 CS. However, a randomized, double-blind Phase III COVACTA trial failed to reveal a significant reduction in mortality by using tocilizumab in COVID-19 (NCT04320615) (5), mandating further (re)search for additional key players in the CS pathogenesis.

The COVID-19 is an adaptive dynamic disease that has witnessed the SARS-CoV-2 mutated multiple times since March 2020. It is highly expected that SARS-CoV-2 will persist as an endemic infection, with epidemic peaks (6), as witnessed with the 4th and 5th waves in some countries. Many of the early models for COVID-19 failed to predict many aspects of the disease (7). Part of the issue is that COVID-19 is a non-linear disease. Many molecular studies were carried out to understand COVID-19 initiation and progression. However, such studies faced various challenges, including the curse of dimensionality (where the total number of severely infected patients is relatively low but each patient has a high number of data points) and inability to find optimal solutions across the general problem and thus end up with sub-solutions (local minima) (8). Artificial Intelligence (AI) is designed to find global solutions to multi-dimensional data. In the context of COVID-19, AI offers vital tools to find better predictors. However, AI has a few limitations in biomedical applications, mainly because AI solutions can be skewed by noise and thus requires well-annotated datasets with a clear understanding of the measured parameters. Integrating clinical, radiological and biochemical tests is highly recommended to achieve the ultimate benefit of modeling the disease.

Interestingly, stochastic modeling was previously used to model the human immune response to the yellow fever vaccine (9). Since COVID-19 is linked to immune response, modeling of the SARS-CoV-2 infection have been extensively published on different aspects of the disease, including the immune system using multiple ODEs to model immune cells, antibodies and cytokines (10–13), and on the clinical and radiological data (14–16). A few models on cytokine release syndrome in other diseases were also created (17–19). Investigating the immune response signature in COVID-19 yielded various biomarkers in different studies. A previous retrospective analysis suggested IL-6, IL-8 and TNF-α as independent predictors of patient survival (20). More recently, Perreau et al., 2021 suggested hepatic growth factor (HGF) and CXCL13 as predictors of severity and mortality of COVID-19 (21).

In the current study, we hypothesized that integrative analysis of pro-inflammatory, anti-inflammatory cytokines and checkpoint markers in addition to key clinical, biochemical, and radiological parameters could predict COVID-19 outcomes with higher predictive accuracy than individual parameters. Guided by the transcriptomics analysis of nasopharyngeal swabs, we curated a panel of 24 cytokines to be assayed using a multiplex assay with high intra- and inter-assay precision to reflect the immune response in our model, using a small amount of serum. Added to the 24 entries of cytokine levels, we also included 63 entries of clinical, biochemical and radiological parameters of well-characterized patients. In this study, we are introducing our clinically applicable integrative model as a predictive tool for COVID-19 severity and sequelae that will hopefully help guide clinical decision and management strategies. In addition, the study highlights potential therapeutic targets *via* identifying key players in the cytokine storm.

## 2 Patients and Methods

### 2.1 COVID-19 Patients’ and Healthy Controls’ Criteria

Nasal swab samples were collected from 50 COVID-19 patients (10 Asymptomatic, 11 mild, 13 moderate, and 16 severe patients; SARS-CoV-2 infections is confirmed by PCR), in addition to 11 healthy donors, at Rashid Hospital in Dubai, following the approval of the ethical committee at Dubai Health Authority (DSREC-04/2020_09). All patients were recruited between February-March 2020, and hence did not receive COVID-19 vaccine.

Peripheral venous blood samples of 37 COVID-19 patients were collected following the approval of the ethical committee at Rashid Hospital in Dubai (DSREC-04/2020_19), in addition to 40 healthy controls. All the included patients were recruited between June-July 2020, and hence did not receive COVID-19 vaccine.

Patients were classified into the respective group severity as follows: (A) Mild-moderate: no or mild pneumonia, (B) severe: patients with at least one of the following symptoms: shortness of breath (breathing rate ≥ 30/min), SaO2 at rest ≤ 93%, partial pressure of oxygen in arterial blood (PaO2)/inspired oxygen fraction (FiO2) ≤ 300 mmHg, or lung infiltrates > 50% within 24 to 48 h. Clinical and biochemical data were collected. Also, computed tomography (CT) imaging was performed, followed by an assessment using the COVID-19 Reporting and Data System (CO-RADS) as a standardized assessment of pulmonary involvement of COVID-19 (22). The Co-RAD categories correspond to the corresponding level of suspicion of pulmonary involvement in COVID-19. 0= scan is technically insufficient to assigning a score; 1=normal or non-infectious; 2= typical for other infection but not COVID-19; 3 = features are compatible with COVID-19 but also other diseases; 4 = highly suspicious for COVID-19; 5 = typical for COVID-19; and 6 = RT=PCR positive for SARS-COV-2.

The healthy controls (age: 47.18 ± 16.6 years, 24 males and 16 females, BMI: 25.9 ± 3.11 Kg/m^2^) were filtered from an initial cohort of 150 controls to include only those with a non-obese BMI and normal HbA1c to avoid having any confounding factors such as obesity or prediabetes.

### 2.2 Whole Transcriptome and Bioinformatics Analysis of Nasal Swab Samples From COVID-19 Patients and Healthy Controls

One ng of RNA of each sample was analyzed using targeted whole RNA-seq with AmpliSeq whole transcriptome on S5 system (Thermo Fisher Scientific), and RNA-seq data were analyzed using the Ion Torrent Software Suite version 5.4. Alignment was carried out using the Torrent Mapping Alignment Program (TMAP), as described in (23).

The expression counts of the nasal swap samples of COVID-19 patients and healthy controls were normalized across samples using the DESeq2 normalization method. Differentially expressed genes between each of the severity groups against the healthy control group were identified using the Bioconductor package DESeq2. Differentially expressed genes with adjusted p-value <0.05 and fold change >2 or <0.5 were considered statistically significant. The adjusted p-value was calculated using false discovery rate (FDR) according to Benjamini Hochberg method (24).

### 2.3 Bioinformatics Analysis of Publicly Available COVID-19 Whole Blood RNA-Seq Dataset

In addition, whole blood bulk RNA sequencing data (Normalized counts) deposited by Bernardes et al. (25) were retrieved from: https://github.com/Systems-Immunology-IKMB/COVIDOMICs/tree/main/TF_enrichment/TF_enrichment_analysis-main/data. The dataset included samples from 42 COVID-19 patients (12 asymptomatic, 11 mild, 6 complicated, 4 complicated incremental, 6 complicated hyper-inflammatory, and 3 critical patients), in addition to 14 healthy donors. Statistical significance of the differential expression of cytokines between the disease severity groups was analyzed using one-way ANOVA with *post hoc* Tukey’s multiple comparisons test in Graph Pad Prism (version 5.01). A p < 0.05 was considered statistically significant.

### 2.4 Collection of COVID-19 Patients’ Blood Samples

Peripheral Venous blood samples of 37 COVID-19 patients were collected following the approval of the ethical committee at Rashid Hospital in Dubai (DSREC-04/2020_19). Ethylene-diamine-tera-acetic Acid (EDTA) containing tubes were used to collect the blood samples. Then, serum was separated for the cytokine assays Forty blood samples were obtained from before the first case of COVID-19 infection in the UAE (MO-HAP/DXB/SUBC/No.14/2017).

### 2.5 Cytokine Assay

Given the results of previous steps, various cytokines of significance were assessed in the sera (50ul sample) of the COVID-19 patients and healthy controls using the Human Immunotherapy Magnetic Luminex Performance Assay 24-plex Fixed Panel (R&D systems, USA). The assessment was done using the Bioplex-200 system (Biorad, USA). A list of the curated cytokines is provided in **Supplementary Table S1**.

### 2.6 Statistical Analysis

Groups with different severity were compared after testing normality (Kolmogorov-Smirnov and Shapiro- Wilk tests). If the p-value is <0.05, non-parametric tests were used (Mann Whitney for comparing two groups, or Kruskal-Wallis to compare more than two groups). We grouped the mild and moderate as (non-severe). Of all variables, only age, BMI and platelet count followed normal distribution where the unpaired t-test was used. Power calculation was performed based on Wei et al. (26), setting the statistical power at 0.8, α = 0.05, and using the mean values of different cytokine levels in mild-moderate versus severe cases. The minimum number required in each group was estimated to be 15. The statistical package SPSS (v.28) was used for statistical analyses.

### 2.7 Machine Learning

Machine learning was used to reduce the set of clinical parameters and identify the optimal set of parameters to stratify the patients according to the different aspects of COVID-19 pathogenesis. A mixture of unsupervised hierarchical and K-means clustering analyses were performed in R (code in the **Supplementary Material**) to assess the separation of COVID-19 cases according to the blood protein expression levels of cytokine quantified using BIO PLEX-200. *k* = 6 was used as the number of clusters for the k-means clustering analysis. The k-means by storing all the labeled examples, and using them directly for inference on new data.

### 2.8 Mathematical Modeling

Mathematical modeling was used to identify key cytokines and biochemical markers that can stratify the clinical parameters collected in the study. The mathematical modeling was carried out using two models that were integrated subsequently. The first is ANOVA multivariate model with Bonferroni’s multiple testing. This is used to identify the variables that are significantly different amongst the various compared patients’ groups as well as different parameters denoting severity (e.g., mechanical ventilation, radiological findings, complications, e.g. liver injury).

The second model is the Stepwise linear regression model. This dynamic method systematically reduces the set of parameters (e.g., cytokines, biochemical parameters), depending on the significant interaction between the variables. The ANOVA multivariate model with Bonferroni’s multiple testing and the Stepwise linear regression model. Both models were applied to a combination of categorical (e.g., disease severity, oxygen support) and continuous data (e.g., protein expression and level of biochemical markers). Two-sided p <0.05 were considered to be statistically significant. ROC analysis was performed to assess the predictive efficacy of the predictors identified from the two mathematical models.

Different parameters were measured to check the model accuracy. if a is true positive, b is false positive, c is false negative, and d is true negative, the sensitivity was calculated as [a/(a+c)]×100; specificity as [d/(b+d)]×100; positive predictive value as [a/(a+b)]×100; and negative predictive value as [d/(c+d)]×100. Positive likelihood ratio was calculated as Sensitivity/(1-Specificity); negative likelihood ratio as (1- Sensitivity)/Specificity (26, 27).

## 3 Results

### 3.1 Nasopharyngeal Samples Identify Cytokines as Top Upregulated DEGs and Signaling Through Cytokines as Top Upregulated Pathway

**Figure 1:**
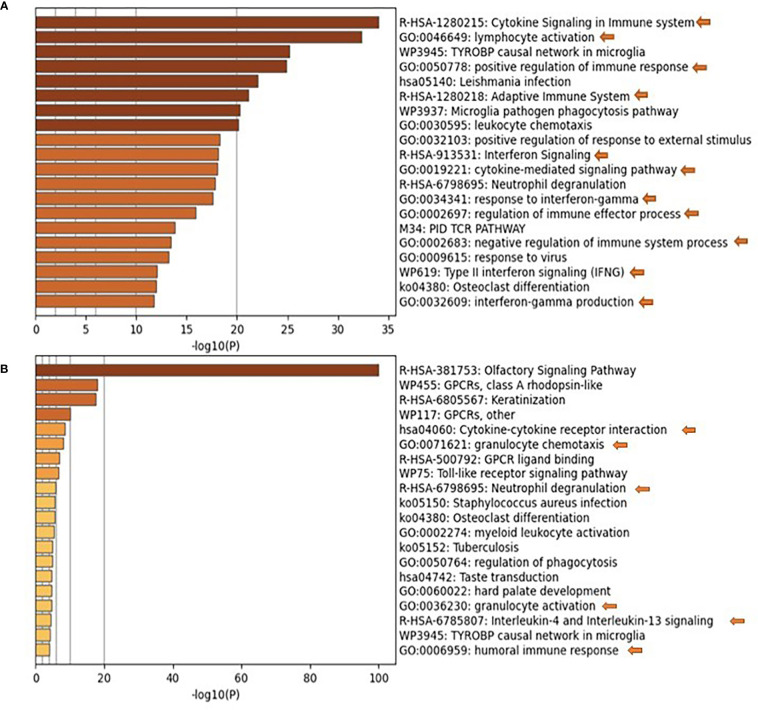
Pathways Enrichment is the nasopharyngeal swab samples of moderate and severe COVID-19 patients. Functional clustering and pathway analysis of the significantly upregulated genes in the nasopharyngeal swab samples collected from **(A)** moderate and **(B)** severe COVID-19 patients in comparison to healthy patients. DEGs were identified using DESeq2 algorithm; the genes were filtered according to adjusted p-value of <0.05 and fold change >2 or <0.5. The functional clustering analysis was performed using Metascape; p-value cut-off for pathways inclusion was <0.01.

**Figure 2:**
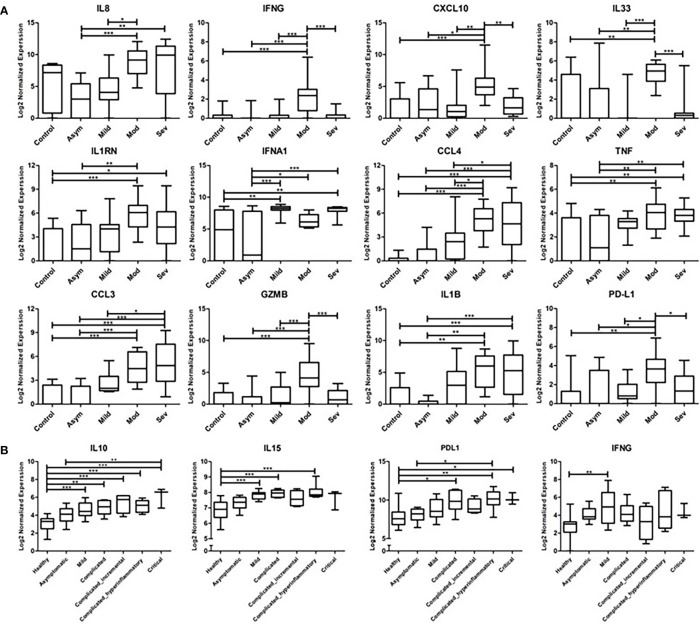
Transcriptomics Analysis of nasopharyngeal swab samples and whole blood samples from COVID-19 patients. **(A)** Gene expression of cytokines and inflammatory mediators from the nasopharyngeal swap RNA-seq data compared across the different severity groups of COVID-19 cases (asymptomatic, mild, moderate, and severe) in reference to the non-COVID-19 control group. The data represented as log 2 normalized expression, where the normalized was performed using DESeq2 normalization approach across all the examined samples. **(B)** Gene expression of cytokines and inflammatory mediators from the whole blood RNA-seq dataset, compared across the different severity groups of COVID-19 cases (asymptomatic, mild, complicated, and critical) in reference to the non-COVID-19 control group. The data represented as log 2 normalized expression. * represents p-value < 0.05; ** represents p-value < 0.01; *** represents p-value < 0.001; analyzed using one-way ANOVA with *post hoc* Tukey’s multiple comparisons test.

Previous studies on nasopharyngeal swab samples were highly insightful on shifts in the immune landscape in association with COVID-19 (28), in contrast to the transcriptomic signature associated with different types of respiratory infections (29). However, general shifts in transcriptomic profiles associated with COVID-19 severity warranted further dissection and biological validation. Therefore, we carried out a transcriptomics analysis of nasopharyngeal samples collected from asymptomatic, mild, moderate, and severe patients using samples from healthy donors as a reference (**Table 1**). The upregulated transcriptome was significantly enriched in cytokine signaling and immune response pathways in moderate and severe COVID-19 patients (**Figure 1** and **Supplementary Figure S1**), with several cytokines being in the top 100 DEGs. Our analysis revealed the significant upregulation of genes expressing IFN-γ, CXCL10, IL-33, Granzyme-B, and PD-L1 in moderate COVID-19 patients only and IL-8, IL-1Ra, IFN-α, CCL4, TNF, CCL3, and IL-1ß was in moderate and severe COVID-19 patients; in comparison, to healthy donors or asymptomatic patients (**Figure 2A**).

**Table 1:** Demographic and Clinical data of the exploratory cohort (COVID-19 patients tested by transcriptomics analysis of nasopharyngeal swabs).

	Asymptomatic (n=10)	Mild (n=11)	Moderate (n=13)	Severe (n=16)	p-value∫
	Mean± SD or N (%)	Mean± SD or N (%)	Mean± SD or N (%)	Mean± SD or N (%)	
Demographics					
Age	36.9 ± 6.64	34.2 ± 6.2	47.6 ± 17.8	60.3 ± 15.6	<0.001
BMI	27.9 ± 1.6	23.3 ± 3.4	27.2 ± 4.5	29.5 ± 5.3	0.015
Gender					
Females	0	3 (27.3)	3 (23.1)	2 (12.5)	0.472
Males	19+0 (100)	8 (72.7)	10 (76.9)	14 (87.5)	
Smoking	0 (0)	5 (45.5)	1 (0.08)	0 (0)	<0.001
Symptoms					
Fever	0 (0)	6 (54.5)	12 (92.3)	16 (100)	<0.001
Cough	0 (0)	6 (54.5)	13 (100)	16 (100)	<0.001
Diarrhea	0 (0)	0 (0)	4 (30.8)	3 (18.8)	<0.001
Dyspnea	0 (0)	2 (18.2)	10 (76.9)	14 (87.5)	<0.001
Loss of smell	0 (0)	5 (45.5)	8 (61.5)	7 (43.8)	<0.001
Nausea/Vomiting	0 (0)	3 (27.3)	3 (23.1)	4 (25)	<0.001
Oxygen supplement					<0.001
Nasal canula, NIV, HFO, Mask	0 (0)	0 (0)	8 (61.5)	3 (18.8)	
Mech Ventilation	0 (0)	0 (0)	1 (0.08)	13 (81.3)	
ICU admission	0 (0)	0 (0)	6 (46.2)	16 (100)	<0.001
Fatality	0 (0)	0 (0)	0 (0)	4 (25)	0.001

∫ Non-parametric tests for continuous variables (Age and BMI) were used (Kruskal-Wallis H), as both were not normally distributed within individual groups. Assessment of severity: Mild to moderate is defined as no or mild pneumonia. The severe type was defined as patients with at least one of the following symptoms: shortness of breath (breathing rate ≥ 30/min), SaO2 at rest ≤ 93%, partial pressure of oxygen in arterial blood (PaO2)/inspired oxygen fraction (FiO2) ≤ 300 mmHg, or lung infiltrates > 50% within 24 to 48 h. Eleven age- and gender-matched healthy controls (age = 27.9± 7.3 years, 9 males and 2 females) were included. N/A, not available.

To investigate whether the upregulation of these cytokines and inflammatory mediators is localized or systemic, the COVID-19 patients whole blood RNA-seq publicly available dataset (25) was analyzed. The analysis revealed the significant upregulation of IL-10 in mild, complicated and critical cases; IL-15 in mild and complicated cases; PD-L1 in complicated and critical cases; and IFN-γ in mild cases (**Figure 2B**).

### 3.2 Cross-Validation of Cytokines Using Bio-Plex

#### 3.2.1 Recruited Patients

Based on previous findings and existing knowledge from previous publications, we examined the association of these cytokines with different clinical aspects of COVID-19 pathogenesis in a new cohort of 37 patients (on day 0-5 of admission, followed up for 4 weeks), and 40 age- and gender-matched healthy controls [Out of initial 150 control subjects, we selected 40, with a non-obese BMI (mean= 25.9 ± 3.11 Kg/m^2^) and normal HbA1c (<5.8%), to avoid having any confounding factors that may affect the cytokine levels, such as obesity or prediabetes]. Patients’ clinical data is provided in **Table 2**.

**Table 2:** Demographic, Clinical and laboratory data of the validation cohort (COVID-19 patients tested for cytokine).

Demographics	Mild to moderate (n=20)	Severe (n=17)	p-value
Age (Mean ± SD)	51.4 ± 15.39	52.59 ± 12.69	0.802
Weight (Mean ± SD)	76.35 ± 15.96	74.35 ± 12.03	0.706
Height (Mean ± SD)	166.75 ± 12.28	165.09 ± 10.8	0.712
BMI (Mean ± SD)	27.7 ± 5.06	27.78 ± 6.42	0.970
Females	7/20 (35)	1/17 (5.9)	0.048
Males	13/20 (65)	16/17 (94.1)	
**Blood type [N (%)]**			0.64
A+	2 (10)	1 (5.9)	
B+	4 (20)	5 (29.4)	
AB+	1 (5)	0 (0)	
AB-	9 (45)	1 (5.9)	
O+	0	9 (52.9)	
O-	1 (5)	0 (0)	
**Clinical Presentation**			
**Symptoms [N (%)]**			0.471
Fever	14 (70)	8 (47.1)	
Cough	10 (50)	8 (47.1)	
Diarrhea	3 (15)	1 (5.9)	
Dyspnea	6 (30)	4 (23.5)	
Confusion	1 (5)	0 (0)	
Nausea/Vomiting	2 (10)	0 (0)	
**Complications**			
None	15 (75)	8 (47.0)	0.118
Thromboembolic event	5 (25)	1 (5.9)	
Hepatic failure	1 (5)	0 (0)	
Renal insufficiency	0	6 (35.2)	
Bacterial co-infection	0	5 (29.4)	
Fungal co-infection	0	5 (29.4)	
**Radiology**			
**X-ray finding [N (%)]**			0.299
None	3 (15)	1 (5.9)	
Consolidation	10 (50)	10 (58.8)	
Ground glass opacities	4 (20)	1 (5.9)	
Pneumothorax	1 (5)	1 (5.9)	
**CORAD score [N (%)]**			0.47
1	2 (10)	2 (11.8)	
2	1 (5)	0 (0)	
4	1 (5)	0 (0)	
6	2 (10)	6 (35.3)	
**Lab Investigations (Mean ± SD)**			
ANC (10^3/µL)	6.57 ± 2.95	13.1 ± 7.83	0.055
ALC (10^3/µL)	1.70 ± 1.07	3.81 ± 3.65	0.242
ANC/ALC (ratio)	6.44 ± 6.06	12.47 ± 14.23	N/A
CRP (mg/L)	38.14 ± 56.31	130.55 ± 126.64	0.001
Creatinine (mg/dL)	0.78 ± 0.21	1.38 ± 1.07	0.006
ALT (U/L)	117.57 ± 180.82	115.14 ± 226.6	0.701
AST (U/L)	87.47 ± 131.72	188 ± 308.18	0.005
D-Dimer (µg/mL)	1.43 ± 2.73	2.87 ± 3.18	<0.001
Ferritin (ng/mL)	568.28 ± 505.40	1468.19 ± 1297.54	0.004
PT (secs)	14.65 ± 1.43	15.49 ± 2.28	0.367
aPTT (secs)	39.77 ± 6.25	45.44 ± 9.27	0.041
LDH (U/L)	359.65 ± 219.76	597.79 ± 262.56	0.005
BUN (mg/dL)	19.31 ± 8.21	67.54 ± 66.79	<0.001
Albumin (g/dL)	3.32 ± 0.46	2.83 ± 0.93	0.04
Bilirubin (mg/dL)	0.86 ± 1.04	0.57 ± 0.36	0.490
Hb (g/dL)	12.01 ± 2.37	11.48 ± 2.48	0.166
Platelets (10^3/µL)	269.50 ± 109.85	287.47 ± 137.72	0.445
WBC (10^3/µL)	9.17 ± 3.38	17.46 ± 8.8	0.008
**Management**			
Azithromycin	1(5)	0 (0)	0.63
Clexane	12(60)	4 (23.5)	0.157
Corticosteroids	6(30)	8 (47.1)	0.97
Favipiravir	6 (30)	5 (29.4)	0.16
Hydroxychloroquine	15 (75)	10 (58.8)	0.59
Interferon-1ß	5 (25)	3 (17.6)	0.24
Kaletra (Lopinavir/ritonavir)	12 (60)	14 (82.4)	0.07
Tocilizumab	2 (10)	6 (35.3)	0.35
Received medications*	18 (90)	15 (88.2)	
**Pressor support**	2 (10)	13 (76.5)	<0.001
**Oxygen supplement**			0.001
Room air	4 (20)	0 (0)	
Oxygen mask, Nasal canula, HFO, NIV	15 (75)	0 (0)	
Mech Ventilation	0	17 (100)	
**Fatality**		**N (%)**	0.374
Died	1 (5)	6 (35.2)	
Discharged from the hospital	19 (95)	11 (64.7)

*Four patients (1 mild, 1 moderate, and 2 severe cases were considered untreated as the samples were withdrawn on the day of admission). Assessment of severity: Mild to moderate is defined as no or mild pneumonia. The severe type was defined as patients with at least one of the following symptoms: shortness of breath (breathing rate ≥ 30/min), SaO2 at rest ≤ 93%, partial pressure of oxygen in arterial blood (PaO2)/inspired oxygen fraction (FiO2) ≤ 300 mmHg, or lung infiltrates > 50% within 24 to 48 h. Forty age- and gender-matched healthy controls (age = 47.18± 16.66 years, 24 males and 16 females) were included. The selected healthy controls had a normal BMI and HBA1c ranges to avoid having any confounding factors such as obesity or prediabetes. ALC, Absolute lymphocytic count; ALT, alanine aminotransferase; ANC, Absolute neutrophil count; AST, aspartate aminotransferase; BUN, Blood Urea Nitrogen; CRP, C-reactive protein; GGT, γ-glutamyl transferase; Hb, hemoglobin; LDH, lactate dehydrogenase; N/A = not available; PT, prothrombin time; PTT, partial thromboplastin time; WBC, White blood cell count.

#### 3.2.2 Cytokine Assay

**Figure 3:**
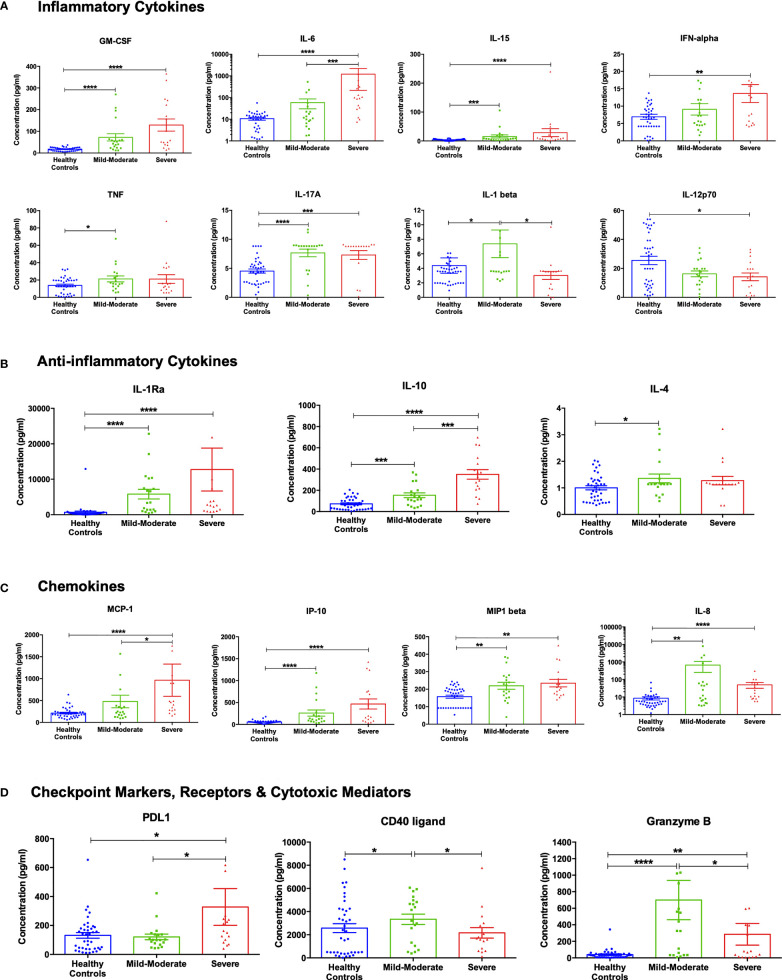
Cytokine assessment in healthy control subjects (n =40), mild-moderate COVID-19 (n= 20) and severe COVID-19 (n=17) patients. **(A)** Inflammatory, **(B)** anti-inflammatory cytokines, **(C)** chemokines, and **(D)** checkpoint markers, receptors and cytotoxic mediators were assessed in mild-moderate and severe COVID-19 patients and their levels compared to healthy controls. Data is expressed as mean ± standard error of mean (SEM). *p<0.05, ** p<0.01, ***p<0.001, and **** p<0.0001.

In view of the transcriptomics analysis, the curated panel included cytokines (inflammatory and anti-inflammatory), chemokines and other immune-related molecules such as checkpoint markers, receptors and cytotoxic mediators (**Supplementary Table S1**). Out of the 24 investigated cytokines, 17 markers showed a differential pattern in COVID-19 patients compared to healthy controls (**Figure 3**).

As shown in **Figure 3A**, the levels of the pro-inflammatory cytokines GM-CSF, IL-6, IL-15, and IFN-α were higher in mild-moderate COVID-19, compared to healthy controls, with a further increase in severe COVID-19. On another note, levels of TNF-α and IL-17A were similarly more elevated in the mild-moderate and severe COVID-19 patients than in healthy controls. Also, IL-1β levels were found to be increased in mild-moderate COVID-19 patients that were restored in severe patients. While the component IL-12p70 showed a reduction in the serum levels of COVID-19 patients with a significant observed decrease in the severe patients’ group, previous studies reported no difference in the plasma levels of IL-12p70 (30). On the other hand, anti-inflammatory cytokines such as IL-1Ra and IL-10 showed a sequential increase in mild-moderate and severe COVID-19, while IL-4 showed a significant increase in mild-moderate COVID-19 (**Figure 3B**).

The chemokines MCP-1 (CCL2) IP-10 (CXCL10) incrementally increased levels in mild-moderate and severe cases (contributors to pulmonary pathogenesis). MIP1β (CCL4) increased equally in mild and severe cases. IL-8 (CXCL8) increased mild-moderate cases and decreased in severe cases (but still significantly higher than normal controls), (**Figure 3C**). As illustrated in **Figure 3D**, PD-L1 was found to be higher in severe COVID-19 than healthy controls or mild-moderate COVID-19. The transmembrane glycoprotein CD40 ligand and the cytotoxic molecule granzyme B showed a significant increase in mild-moderate COVID-19 patients compared to healthy controls. However, they were reduced in the severe patients (but still significantly higher than normal controls).

### 3.3 Exploration of Cytokines Expression Levels in Association With COVID-19 Disease Severity Using Machine Learning Techniques

The protein expression data of the cytokines and inflammatory mediators were further explored with machine learning approaches to identify the optimal set of parameters to stratify the patients according to different aspects of COVID-19 pathogenesis. Initially, unsupervised hierarchical and k-means clustering were used to explore the general impact of cytokines expression on the clustering of the examined COVID-19 patient samples according to disease severity. The result of the unsupervised hierarchical and k-means clustering showed that the collective cytokines panel had little impact on the clustering of the samples according to disease severity, as cases of different degrees of severity were intermingled in both clustering approaches; suggesting an overlap in the signature of some cytokines across the different severity groups. However, the unsupervised clustering gave hints of separation between severe and moderate cases, suggesting that some of the cytokines might have the potential to stratify patients according to disease severity. Therefore, mathematical modeling was carried out to explore further and identify the cytokines that significantly associate with disease severity and other aspects of COVID-19 pathogenesis.

### 3.4 Optimal Parameter Selection Using Mathematical Modeling

To filter out the biological overlap between the severity groups in the data set and identify key cytokines and biochemical markers that can be used to stratify the clinical parameters collected in the study, an approach combining two mathematical models (multivariate ANOVA with Bonferroni’s stringent multiple testing and Stepwise linear regression) were used (**Supplementary Table S2**).

#### 3.4.1 Mathematical Modeling Identifies IL-10 as a Biomarker of Severity

**Figure 4:**
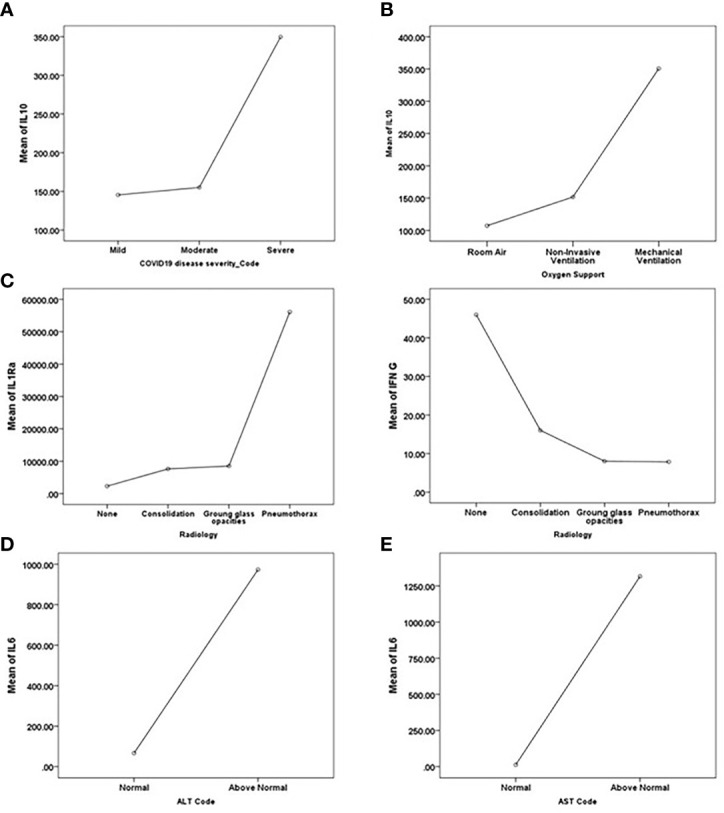
Key driver predictors identified from Multivariate ANOVA with Bonferroni’s stringent multiple testing for **(A)** disease severity, **(B)** the requirement for oxygen support, **(C)** Radiological findings, and **(D, E)** abnormal liver function indicated by **(D)** ALT and **(E)** AST. Means of the predictors’ levels presented as a function of the target variables categories.

Multivariate ANOVA with Bonferroni’s stringent multiple testing was used to determine whether there were statistically significant differences in the expression of particular cytokines and biochemical markers between the COVID-19 severity groups (**Figure 4A**). The analysis revealed that the levels of IL-10, ANC, ALC, CRP, Ferritin, LDH, BUN, and WBCs were significantly higher in severe cases in comparison to mild-moderate cases. The stepwise linear regression model identified IL-10, PD-L1, TNF -α as potential predictors of COVID-19 disease severity. The data from these two mathematical models suggest a panel of cytokines and biochemical markers for stratifying COVID-19 patients according to disease severity, with the circulating marker IL-10 as the driver key marker.

#### 3.4.2 Mathematical Modeling Identifies IL-10 as a Biomarker of Oxygen Support Requirement

A similar analysis was performed to determine the potential association between the level of cytokines and biochemical markers and other aspects of COVID-19 pathogenesis, such as the need for oxygen support. Multivariate ANOVA testing suggested the significant association between the need for oxygen support and the levels of GM-CSF, IL-1β, IL-10, ANC, CRP, Ferritin, LDH, BUN, and WBC. Multivariate ANOVA with Bonferroni’s stringent multiple testing revealed the significant increase in the levels of IL-1β and IL-10 in patients requiring mechanical ventilation as opposed to patients depending on room air; and a significant increase in the levels of ANC, LDH, BUN, and WBCs in patients requiring invasive mechanical ventilation in comparison to patients requiring non-invasive forms of oxygen support (e.g., nasal cannula, high flow oxygen mask, and non-invasive positive pressure ventilation) (**Figure 4B**). The stepwise linear regression model identified IL-10 as a potential predictor of the need for oxygen support. Taken together, both mathematical models suggest IL-10 as a potential marker for the requirement for oxygen support in addition to its potential in stratifying disease severity.

#### 3.4.3 Mathematical Modeling Identifies IL1Ra and IFN-γ as Biomarkers of COVID-19-Specific Radiological Findings

Analysis of the association between chest X-ray (CXR) findings and the levels of cytokines and biochemical markers using multivariate ANOVA with Bonferroni’s stringent multiple testing revealed the upregulated levels of IFN-γ and PD-L1 in normal cases as opposed to patients presenting with consolidation or ground-glass opacities (**Figure 4C**). On the other hand, IL-1Ra, IL-6, MCP-1, and D-dimer levels were elevated in cases presenting with pneumothorax compared to normal cases or cases presenting with consolidation or ground-glass opacities. Stepwise linear regression analysis proposed IL-1Ra, IFN-γ, and PD-L1 as potential predictors of radiological findings. Stepwise linear regression analysis of CORADs reports suggested IL1Ra and IFN-γ as predictors of radiological findings, further cross-validating the CXR analysis results. Taken together, these data suggest that IL-1Ra and IFN-γ might potentially be used to stratify patients according to radiological findings; IL-1Ra as a potential marker for the development of pneumothorax and IFN-γ as a potential marker predicting the absence of COVID-19 related chest abnormalities.

#### 3.4.4 Mathematical Modeling Identifies IL-6 and Granzyme B as Biomarkers of Liver Injury and Dysfunction

The stepwise linear regression model identified IL-6 and granzyme B as potential predictors of liver injury and dysfunction (indicated by an elevation in the levels of ALT and/or AST). IL-6 and granzyme B levels were elevated in cases with abnormal ALT levels (**Figure 4D**), while IL-6 was elevated in patients with abnormal levels of AST (**Figure 4E**).

Intriguingly, Multivariate ANOVA with Bonferroni’s multiple testing revealed the significant reduction in the protein expression level of IL-6 in patients that received COVID-19 treatments (e.g., tocilizumab, lopinavir/ritonavir, favipiravir) in comparison to untreated patients. Treatment status associated significantly with reduced levels of other markers, including IL-1Ra, MCP-1, PD-L1, ALT, D-Dimer, and Albumin.

### 3.5 Validation of Predictor-Based Stratification of Severity Groups in COVID-19

**Figure 5:**
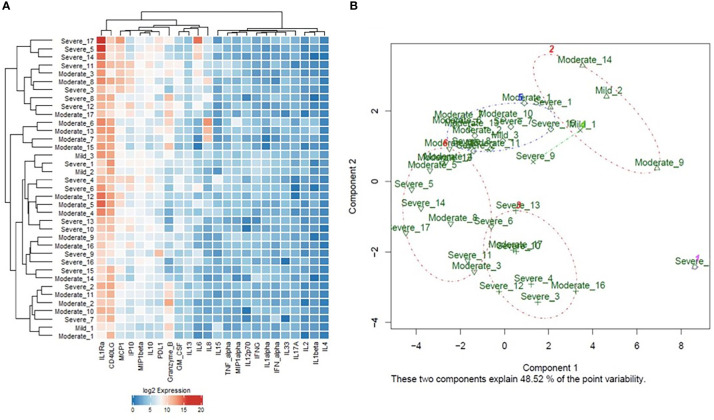
**(A)** Heat map representation of the unsupervised hierarchical clustering and **(B)** Principal Component Analysis (PCA) plot representation of the k-means clustering analysis of cytokines protein expression in the blood samples of COVID-19 patients of different degrees of severity (3 mild, 17 moderate, and 17 severe). ROC analysis of the predictive capacity of the cytokines (AUC=0.93 ± 0.037, 95% CI=0.86-1, p<0.0001). ROC analysis of the predictive capacity of the biochemical markers (AUC=0.98 ± 0.02, 95% CI=0.94-1, p<0.0001), identified using the mathematical models to stratify COVID-19 patients according to disease severity.

Cytokines proposed as predictors of disease severity by the two mathematical models were used for model reduction to enhance patients’ clustering. The supervised hierarchical and k-means clustering revealed an enhanced clustering of patients according to disease severity (**Figure 5A, B**); where severe cases were enriched in the cluster indicated by the red brackets in the heat map (**Figure 5A**) and clusters 1, 4, and 6 in the k-means PCA plot (**Figure 5B**). Moreover, ROC curve analysis was used to assess the predictive efficacy of the predictors identified using the mathematical modeling approach to stratify patients according to disease severity. Analysis of the collectively identified cytokines from the two mathematical models (IL-1-α, IL-4, IL-10, IL-13, PD-L1, TNF-α) revealed a significant predictive efficacy with an area under the curve (AUC) value of 0.935. Similarly, assessment of the predictive capacity of the collective biochemical markers identified using multivariate analysis (ANC, Ferritin, LDH, BUN, and WBC) confirmed a significant predictive efficacy with an AUC value of 0.981, **Supplementary Figure S2**.

ROC analysis of each cytokine and biochemical markers was performed to suggest potential cut-off values with high sensitivity and specificity and significantly high predictive efficacy accordingly. The analysis revealed a cut-off value of 204.5 pg/ml for IL-10, 117.27 pg/ml for PD-L1, 724.0 ng/mL for ferritin, 325.0 U/L for LDH, 10.25×10^3^/μL for WBC, and 28.27mg/dL for BUN. Cutoff values of the identified predictors for other variables are listed in **Supplementary Table S3**.

### 3.6 Mapping of Significantly Differentiated Cytokines on the KEGG Pathways

**Figure 6:**
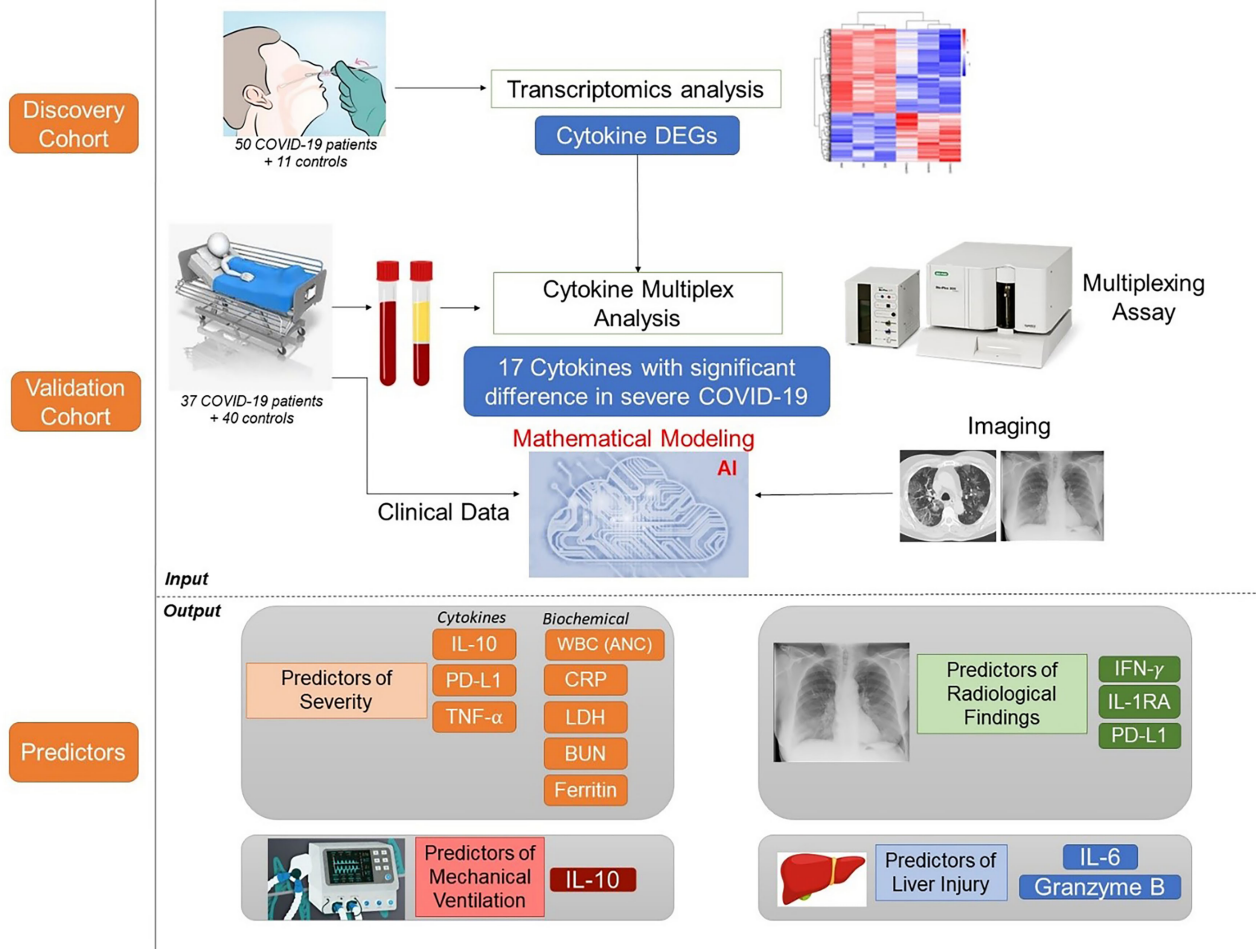
Graphical Abstract of the work flow and the main results.

We further mapped the “predictor” cytokines on several immune-related KEGG pathways, as well as the SARS-CoV2 entry pathway. Of interest, several significantly elevated cytokines in severe COVID-19 patients are remarkable key players along Natural Killer (NK) cell-mediated cytotoxicity pathway (**Supplementary Figure S3**). **Figure 6** summarizes the workflow and the main results.

## 4 Discussion

In this study, we aimed to predict the outcome of COVID-19 using a non-linear mathematical model of serum cytokine changes, in addition to clinical, biochemical, and radiological parameters. Compared to previous studies, we used AI techniques to integrate data from different modalities (clinical parameters, biochemical tests, cytokine assays, and radiological data) for the first time. We included 87 parameters as input to our model, covering 24 cytokines classified as pro-inflammatory, anti-inflammatory, chemokines, checkpoint markers, receptors and cytotoxic mediators. Cytokines were selected based on an initial transcriptomics analysis of nasopharyngeal swabs of COVID-19 patients and control subjects. Although the unsupervised hierarchical and k-means clustering showed that the *collective* cytokines panel had little impact on the clustering of the samples, the supervised clustering gave hints of separation between severe and moderate cases, identifying the cytokines with potential predictive value for COVID-19 severity. Taking the initial large number of clinical parameters, biochemical markers and cytokines expression data that would result in a vast number of permutations, machine learning and mathematical modeling were used to filter the data to achieve model reduction and identify the optimum associations to stratify patients according to multiple aspects of COVID-19 pathogenesis. Given the emerging new variants of the virus, our modeling strategy can be applied to different datasets to predict outcomes in new cases of COVID-19 and identify fundamental immune-mediated mechanisms and potential therapeutic targets for such new variants.

Interestingly, in our study, there was a significant upregulation of IL-10 and IL-15, consistently associated with disease severity in both investigated COVID-19 whole blood dataset and our cytokine assays, suggesting the potential utility of these predictive cytokines as circulating biomarkers of severity. IL-10 was reported to contribute to the suppression of the immune system, viral control, and disease severity (31), and a predictor of poor outcomes in COVID-19 patients (32–34). This was possibly linked to its role as an anti-inflammatory cytokine, released as negative feedback in response to the rapid accumulation of pro-inflammatory cytokines (33, 34), and aids in alleviating the CS and preventing tissue damage (31).

Therefore, recombinant IL-10 has been suggested by some investigators for treating acute respiratory distress syndrome (ARDS) in COVID-19 patients based on its immune-regulatory and anti-fibrotic functions (32). Moreover, IL-15/IL-15R axis plays a pivotal role in the function of NK cells (35). IL-15 is produced by activated monocytes/macrophages and activates human NK cells through components of the IL-2R in a pattern similar to that of IL-2. IL-15 also induces IL-10 expression by the NK cells, enhancing its cytotoxic effect. The effect of IL-10 on NK cells is mediated through STAT3 signaling according to *in-vitro* studies (36). Furthermore, Wang et al., 2021, deciphered that IL-10 regulates metabolic reprogramming in NK cells, *via* stimulation of the mammalian target of rapamycin complex 1 (mTORC1). In that way, it upregulates glycolysis as well as oxidative phosphorylation in NK cells, thus might enhance the functions of the NK cells (37). Masselli E. et al. reported that the IL-15/IL-15R axis was among the top pathways associated with severe/fatal disease in the viral pandemic gene signature. This may shed some light on the mechanistic role of this axis in the immune NK cell response derangement, which leads to NK cells exhaustion, senescence, apoptosis, and viral persistence (38). Interestingly, severe COVID-19 resulted in an increase of “armed” NK cells containing high levels of cytotoxic proteins such as perforin (39). NK cells are obviously major players in the immune response during COVID-19 infection, but similar to hepatitis virus infections, they may become dysfunctional during severe disease, and their role in organ dysfunction (e.g., liver) requires further investigation (40). It was reported that NK cells might undergo pyroptosis, releasing inflammatory cytokines such as IL-1β (41). However, it is not clear yet whether elevation of IL-15 may activate NK cells, thus contributing to the cytokine burst observed in these patients. In addition, IL-15 was previously suggested to play a role in granulomatous pulmonary diseases through its stimulation of Th-1-driven inflammation (42). Recently, it was also reported to be involved in the development of rapidly progressive interstitial lung disease in polymyositis/dermatomyositis (43). Although reported to enhance NK-cell cytotoxicity, IL-10 elevation may be also a consequence of NK-cell stimulation in an attempt to ameliorate the prevalent hyper-inflammatory state of the CS.

The role of NK cells in COVID-19 infections has not been examined in detail, although it was suggested that they may be important participants (44). In this study we observed that the level of IL-15, a vital cytokine for NK-cell activity, is highly increased, suggesting that NK cells might play a role. It was previously reported that these cells secrete a variety of inflammatory cytokines and chemokines (45), which may contribute to the cytokine storm described in COVID-19 patients.

Several patterns of cytokine changes were identified across the severity levels of COVID-19. Our results showed significant changes in pro-inflammatory cytokines (GM-CSF, IL-6, IL-15, IFN-α, TNF-α, IL-17A) that play a crucial role in the CS (46). The SARS-CoV-2 infection causes local innate immune cells to produce such inflammatory cytokines upon infection of the respiratory epithelial tissue and cause the activation of the adaptive immune cells leading to respiratory epithelial damage (47). This is further supported by activating the inflammasome and NF-κB pathways, inducing the stimulation of several pro-inflammatory genes and immune cell hyperactivation, thus boosting systemic inflammation. In the setting of inflammation, IL-6, which is generated by macrophages and dendritic cells, is known to be a key activator of the JAK/STAT3 pathway (48). Also, IL-6 was reported to contribute to immune cell hyperactivation and target organ dysfunction in COVID-19. On the other hand, the destruction of epithelial cells in the alveolar space caused by SARS-CoV-2 triggers macrophages hyperactivation leading to the CS. IL-6 was found to suppress T lymphocyte activation, that could contribute to lymphopenia in COVID-19 patients. Also, low numbers of T lymphocytes were observed with ICU patients showing high IL-6 and TNF-α serological levels (49–51). Similarly, IL-17 was found to exacerbate lung injury and decrease the survival through the recruitment of neutrophils and stimulation of pro-inflammatory factors (52). GM-CSF also triggers myelopoiesis in order to recruit myeloid cells to the inflammatory sites (53). It was previously suggested as a potential therapy for the COVID-19 CS (47).

Noteworthy, the concurrent elevations in IL-10 and various pro-inflammatory cytokines, and the observed relationship between elevated IL-10 levels and disease severity, suggest that IL-10 is either failing to appropriately suppress inflammation (as observed in other inflammatory conditions (54, 55) or acting in a manner that deviates from its traditional role as an anti-inflammatory molecule, indicating the ability of IL-10 to have different functions under different conditions (32).

The anti-inflammatory IL-4, along with IL-13, mediate the Th2 cell response and M2 polarization, leading to consequent fibrosis and release of growth factors, such as transforming growth factor-β and platelet-derived factor (46, 56). IL-1Ra is known to control the inflammatory immune response by binding to the IL-1R and regulating the production of inflammatory cytokines such as IL-1 and TNF-α (57). In COVID-19 infection, IL-1Ra was suggested to affect the stimulation of pro-inflammatory and antiviral cytokines, where its high level could be an indication of an overactive immune response, thus leading to inflammation-induced tissue damage (34). Controversial patterns were reported regarding IL-4 in previous studies where some reported an increase in peripheral blood/serum of severe COVID-19 patients (30, 46, 58), while others claimed that it did not show any difference (34, 59).

We observed increased levels of chemokines MCP-1 (CCL2), IP-10 (CXCL10), MIP1β (CCL4) and IL-8 (CXCL8) in COVID-19 patients. These chemokines are known to be crucial contributors to pulmonary pathogenesis, such as that observed in COVID-19. CCL2 is known to be released by alveolar macrophages, T cells and endothelial cells in order to induce the migration of inflammatory monocytes and neutrophils along with procollagen synthesis by fibroblasts (60). CXCL10, a known chemoattractant for monocytes, NK and T cells (61), was also reported to play a crucial role in pulmonary neutrophil infiltration (62). Moreover, CXCL10/CXCR3 axis triggers the oxidative burst which promotes exacerbation of the pulmonary inflammation and progression to ARDS (62). CCL4 acts through CCR5 receptor to attract macrophages, dendritic cells, NK and T cells to the site of inflammation (63). Interestingly, the CCR5 antagonist, maraviroc (an antiretroviral medication) was repurposed for moderate to severe COVID-19 (NCT04435522 and NCT04441385). CXCL8 was reported to be responsible for the recruitment, activation, and accumulation of neutrophils (64). Furthermore, it induces the formation of neutrophil extracellular traps (NETs) that further promote inflammation and tissue injury (65). Elevated CXCL8 levels at the time of hospitalization, along with IL-6 and TNF-α, was previously suggested as strong and independent predictors of survival in COVID-19 (20).

PD-L1 was higher in severe COVID-19 patients, whereas the levels of CD40 ligand and granzyme B showed a significant increase in mild-moderate COVID-19 patients, but reduced in severe patients. PD-L1 induces inhibitory signals and apoptosis of CD8+ T cells. This is induced by binding of the pro-inflammatory cytokines to their respective receptors. Hence, the release of IL-6, IL-17, and TNF-α, along with the increased activity of macrophages and neutrophils, cause the increased expression of PD-L1 on the surfaces of immune cells in COVID-19 (66), through the STAT3, PI3K/Akt and NF-κB pathways. CD40L, a costimulatory molecule present on T cells, was found to be released by activated platelets in the serum, that may contribute to pulmonary thrombotic complications observed in COVID-19 as well as being associated with ARDS status (67, 68). Previously, studies have shown that PD-L1 could be a potential predictive factor in various types of cancer (69). The cytotoxic mediator, granzyme B, along with perforin, are the main mediators through which NK cells and cytotoxic T lymphocytes eliminate virally infected host cells, as in COVID-19 infection (70). Interestingly, NK cells from COVID-19 patients exhibit higher levels of granzyme B that is associated with the severity of the disease (71). To re-iterate, our study emphasizes the role of NK cells in COVID-19 infection, an enigma that was not previously resolved. In addition, previous reports revealed that IL-10 and PD-L1 suppress T-cell activity during persistent viral infection (72), thus giving mechanistic insight towards persistent COVID-19 and the potential role of targeting both cytokines to minimize the long-term sequelae of the disease.

Added to combining selective recognized biochemical markers of COVID-19 severity (ANC, CRP, LDH, BUN and ferritin), the triad of elevated serum IL-10, PD-L1 and TNF-α improved the current model accuracy to predict the severity of the disease, through the stepwise linear regression model. The results from these two mathematical models suggest the circulating marker IL-10 as a driving key marker for the stratification of COVID-19 patients according to disease severity. Noteworthy, IL-10 is elevated earlier than IL-6 in COVID-19 patients (32, 34).

Our mathematical model identified IL1-α, IL-4 as negative predictors of severity. As both are involved in adaptive immunity, highlighting its marked derangement in severe COVID-19. In contrast to our results, other reports showed elevated IL1-α in severe COVID-19 that was strongly associated with lung injury (preprint by Liu et al., 2020). Controversial patterns were reported regarding IL-4 plasma levels where some reported an increase in peripheral blood/serum of severe COVID-19 patients (30, 46, 58, 73), while studies show any difference (34, 59).

IL1Ra and IFN-γ were identified in our model, as biomarkers of COVID-19-specific radiological findings. The IL-1 superfamily was previously recognized as a key mediator of inflammation and fibrosis in different organs, with IL-1Ra as an antagonistic cytokine (74). The crucial balance between IL-1β and IL1Ra determines the resultant immune response in many tissues (74). In the severe COVID-19 cases in this study, IL-1β significantly decreased and IL1Ra significantly increased as a part of the marked immune dysregulation. This was associated with specific COVID-19 related radiological findings, as revealed by the mathematical model. IFN-γ mediates immune-mediated damage in acute lung injury (75).

In support of our findings related to IL-6 and granzyme B as biomarkers of liver injury, a recent study demonstrated that IL-6 trans-signaling drives COVID-19-associated hepatic endotheliopathy, which is suggested as a possible mechanism underlying the liver injury (29). Previous reports highlighted the role of NK cells and their enzymes (Granzyme B and perforin) in hepatic immune homeostasis (76). IL-6 was reported to suppress the NK cytotoxicity *in-vitro* and *in-vivo* (77). However, in view of the multiple cytokines affecting the NK cells in the CS context and the elevation of IL-15 (NK stimulator), the effect of IL-6 is surpassed, with a net result of increased granzyme B. Our model shows the high accuracy of liver injury prediction in severe COVID-19, by combining IL-6 and granzyme B as predictors.

We used a stochastic non-linear modeling approach to reduce the dataset for multi-dimensional data and to integrate data from different modalities. The non-linear ODE model is crucial to clearly reflect the dynamics of biological systems (78). To estimate the exact probabilities for biological systems, approaches are mainly based on Monte Carlo sampling (e.g. the Stochastic Simulation Algorithm) (79). To create a dynamic model of CS, Waito et al., 2016 used a nonlinear differential equation model, considering the cytokine production rate in relation to their interactions with one another. They adjusted the model by using the data from a CS mouse model (IFN type 1 receptor KO). Interestingly and concordant to our results, the model revealed that TNF-α, IL-10, IL-6, and MIP-1β, exerted the largest effects on the dynamics of the cytokine storm (17). In the current study, we used non-linear modeling that attempts to identify global solutions to integrate and explore biomarkers that can predict COVID-19 severity (8).

Our study sheds light on key immunological aspects of the COVID-19-CS that seem to significantly differ from the CS occurring in other diseases. Beyond its value as a biological predictive tool, our mathematical analysis poses important questions for future research.

## 5 Conclusions

Predictive modeling in COVID-19 has gained a high value, considering the complexity of the disease. Using a non-linear model for clinical, biochemical, immunological, and radiological data could achieve a high level of prediction accuracy. In our proposed integrative model, we validated a cytokine panel derived from transcriptomics analysis of nasopharyngeal swab samples of COVID-19 patients. Our model advocates the trio of IL-10, PD-L1 and TNF-α as an accurate predictor of severity, in addition to previously recognized ANC, CRP, LDH, BUN and ferritin, whereas IL-1α, IL-4 were negative predictors. IL-10 was shown to be a driving marker and a positive predictor of mechanical ventilation. Moreover, IFN-γ, IL-1Ra were predictors of remarkable radiological findings, whereas high IL-6 and granzyme B were found to predict liver injury in COVID-19 patients.

We identified key cytokines that were consistently associated with severity, like IL-10, an enhancer of NK cytotoxicity, and IL-15, a stimulator of NK cells, Obviously, the modeling methodology can be used to identify key players and predict outcome in new variants of COVID-19.

## Data Availability Statement

The datasets presented in this study can be found in online repositories. The names of the repository/repositories and accession number(s) can be found below: https://figshare.com/articles/dataset/Transcriptome_COVID19_Cytokine_Mathmodel_xlsx/19386194.

## Ethics Statement

The studies involving human participants were reviewed and approved by Approval of the Research and Ethics Committee at Dubai Health Authority (DSREC-04/2020_09) and (DSREC-04/2020_19). The patients/participants provided their written informed consent to participate in this study.

## Author Contributions

Conceptualization: MS-A and RH. Data curation: NE, SH, LS, and MS. Formal analysis: NE, SH, PH, and MS. Funding acquisition: MS-A and RH. Investigation: NE, SH, and IT. Methodology: MS-A, RH, NE, and SH. Project administration: MS-A. Resources: LS, BM, HS, MS, KOH, OA-A, JT, NS, AM, and QH. Software and mathematical modeling: SH. Supervision: RH, BM, HS, and MS-A, Validation: NE. Visualization: NE, SH, and PH. Writing - original draft: NE, SH, IT, MS-A, JT, and RH. Writing - review and editing: HS, AM, and QH. All authors contributed to the article and approved the submitted version.

## Funding

MS-A and RH are funded by the University of Sharjah Research Grants CoV-19 #0304 and CoV-19 #0308.

## Conflict of Interest

The authors declare that the research was conducted in the absence of any commercial or financial relationships that could be construed as a potential conflict of interest.

## Publisher’s Note

All claims expressed in this article are solely those of the authors and do not necessarily represent those of their affiliated organizations, or those of the publisher, the editors and the reviewers. Any product that may be evaluated in this article, or claim that may be made by its manufacturer, is not guaranteed or endorsed by the publisher.
