# Supplementary material for: Identifying Immunological and Clinical Predictors of COVID-19 Severity and Sequelae by Mathematical Modeling
Source: Front Immunol. 2022 Apr 20;13:865845. doi: 10.3389/fimmu.2022.865845 (PMC9067542; doi:10.3389/fimmu.2022.865845)
Supplement: Supplementary file 1 [file DataSheet_1.pdf]

Supplementary Material

A **Asymptomatic vs. Control**

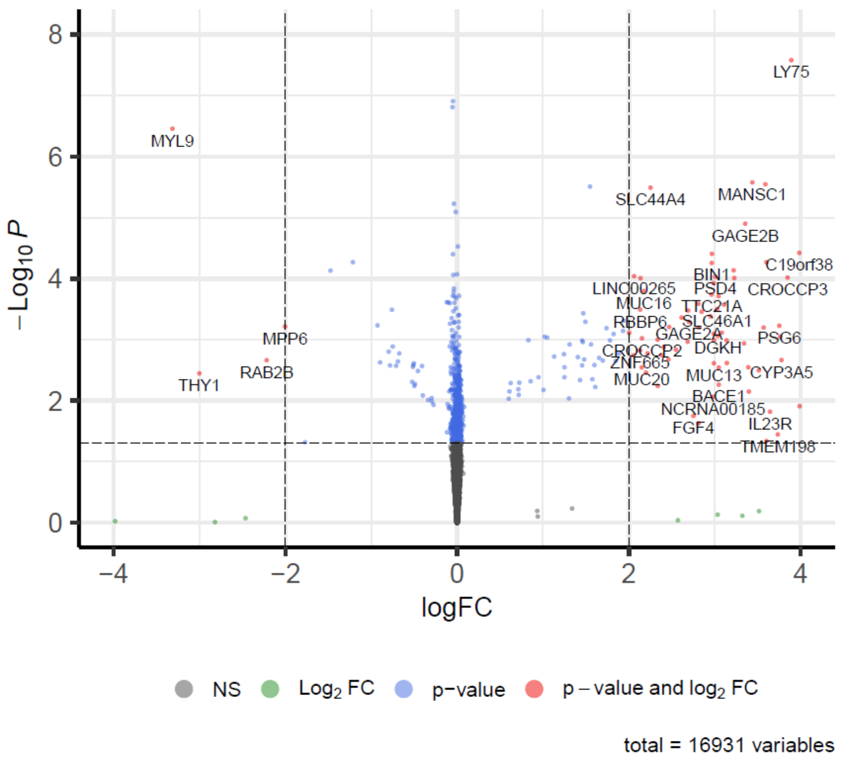

B **Mild vs. Control**

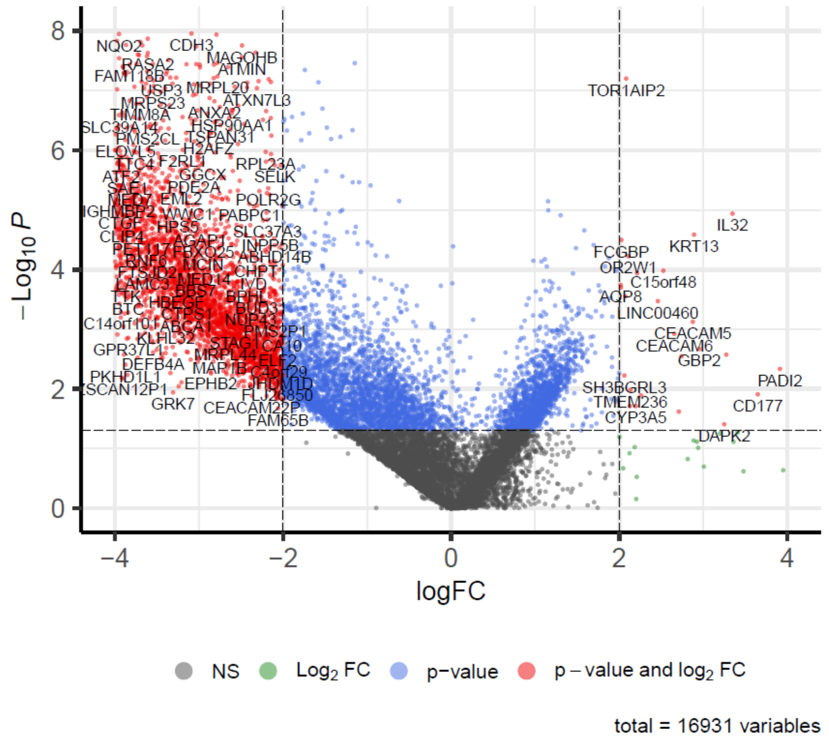

Supplementary Material

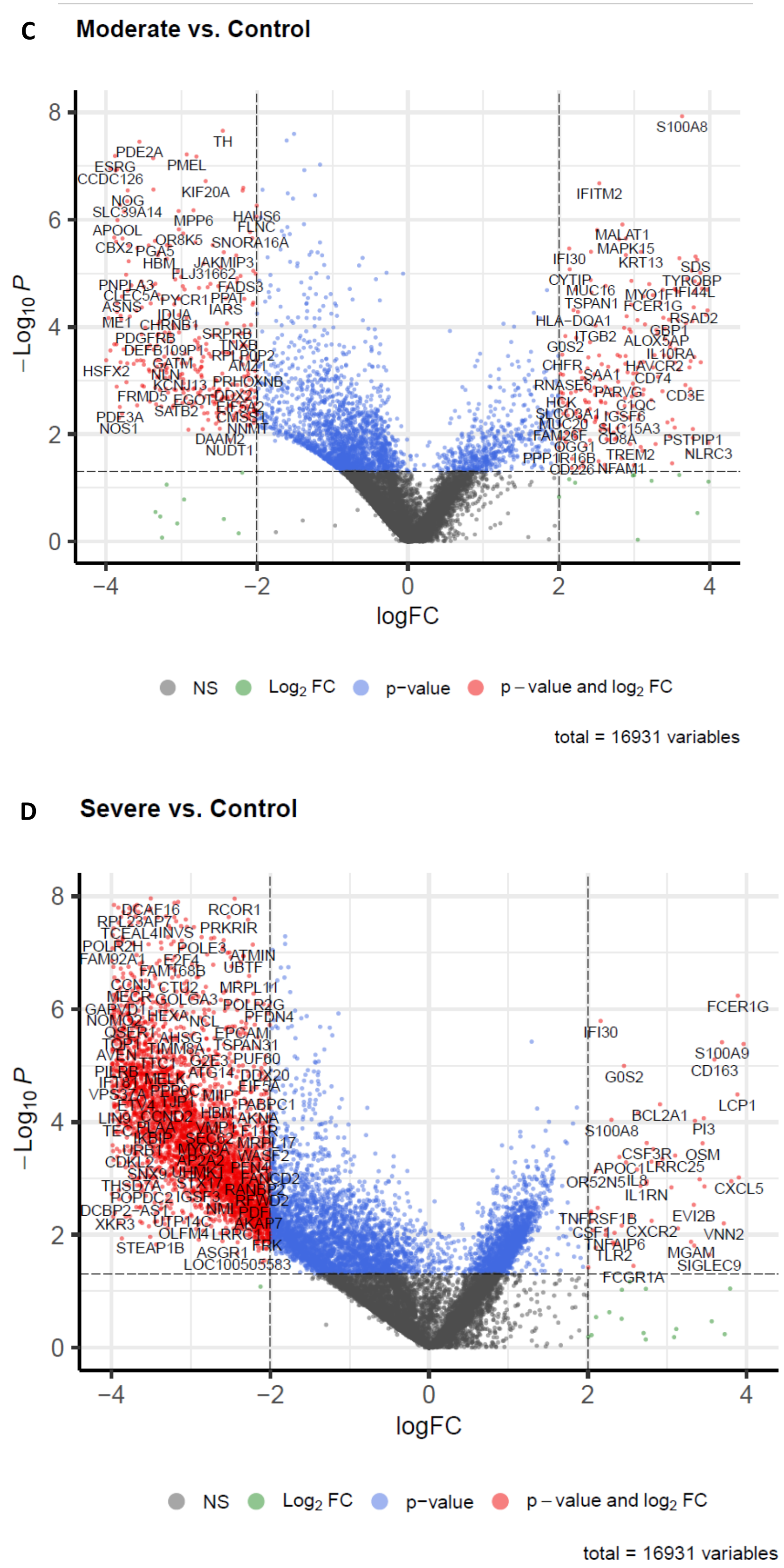

Figure S1: Volcano plots of differentially expressed genes for the transcriptomics analysis of the naso-pharyngeal samples of **A.** asymptomatic, **B.** mild, **C.** moderate, and **D.** severe cases of COVID-19. The differentially expression is presented as a log fold change (log FC).

A

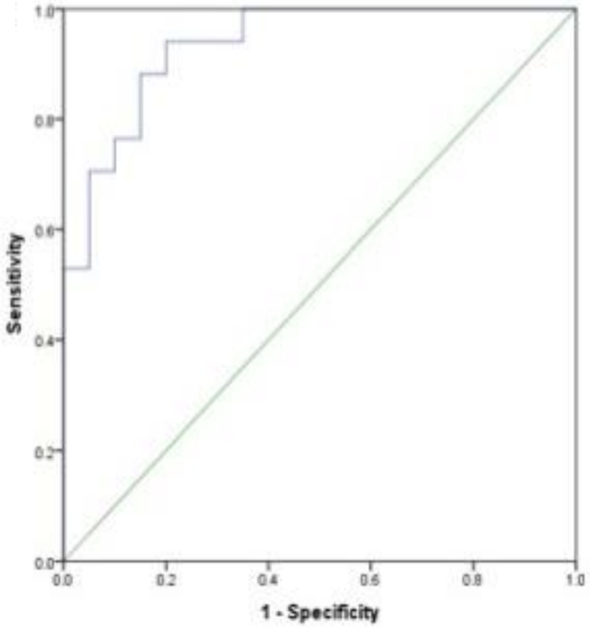

AUC	Std. Error[a]	Asymptotic Sig.[b]	Asymptotic 95% Confidence Interval Lower Bound	Asymptotic 95% Confidence Interval Upper Bound
0.935	0.037	0.000	0.862	1.000

B

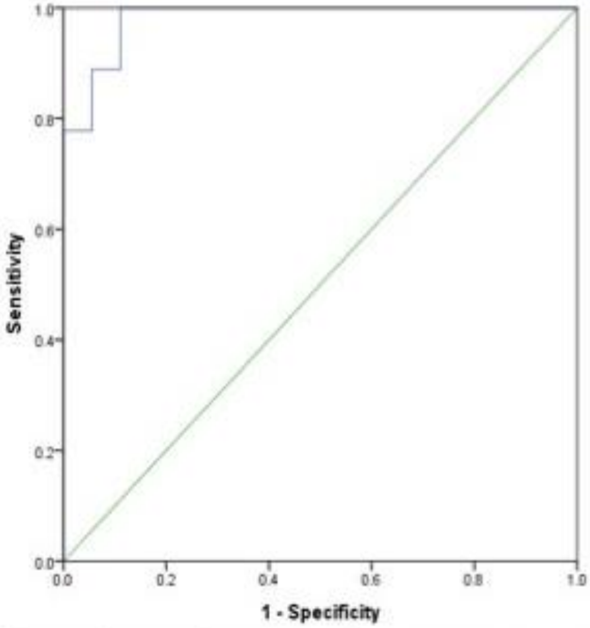

AUC	Std. Error[a]	Asymptotic Sig.[b]	Asymptotic 95% Confidence Interval Lower Bound	Asymptotic 95% Confidence Interval Upper Bound
0.981	0.020	0.000	0.942	1.000

Figure S2. A. ROC analysis of the predictive capacity of the cytokines (AUC=0.93±0.037, 95% CI=0.86-1, p<0.0001). ROC analysis of the predictive capacity of the biochemical markers (AUC=0.98±0.02, 95% CI=0.94-1, p<0.0001), identified using the mathematical models to stratify COVID-19 patients according to disease severity.

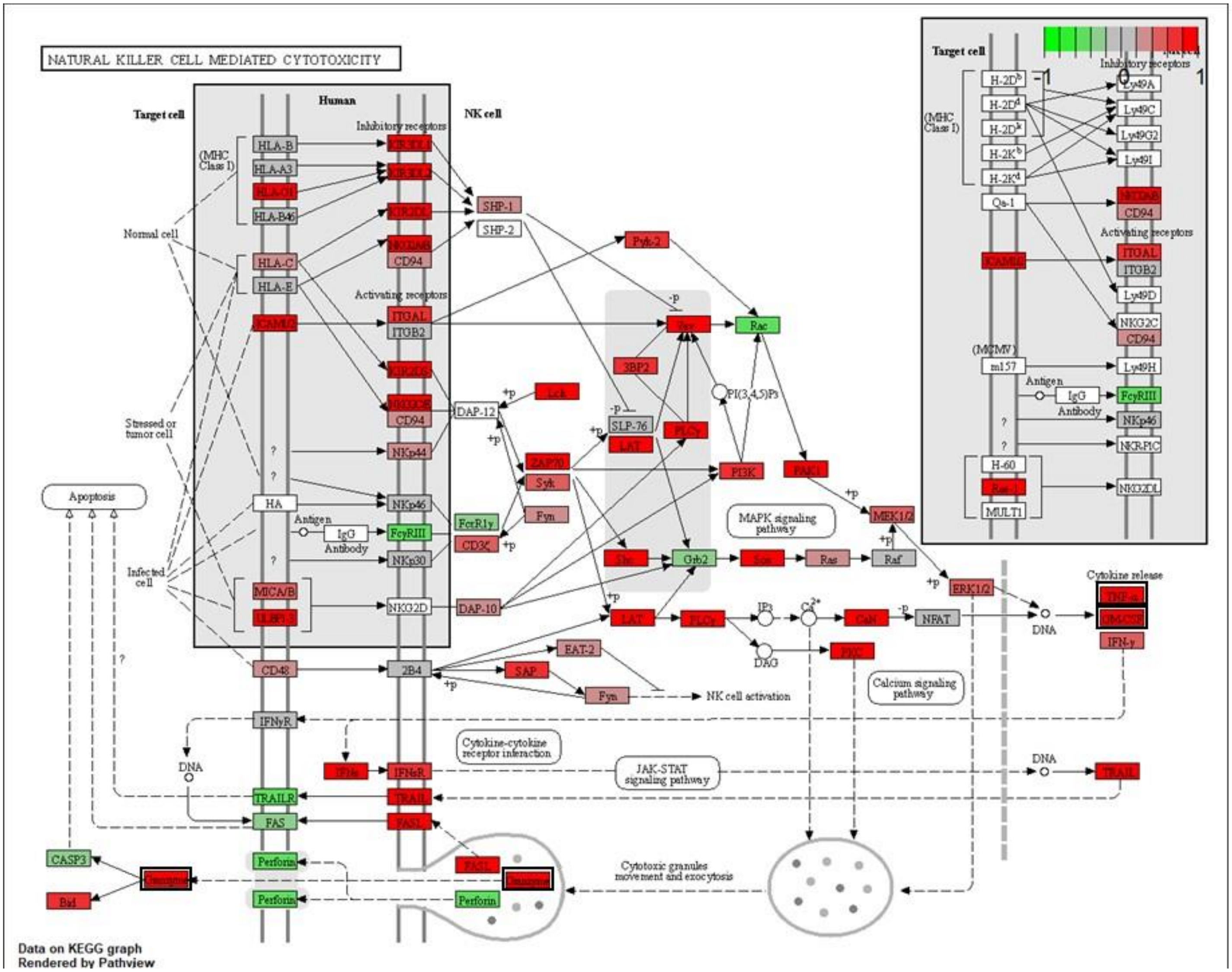

Figure S3. KEGG NK mediated cytotoxicity pathway with mapping of DEG in the dataset (GSE). Black Squares denote cytokines with significant results in both study cohort (nasopharyngeal swabs and serum).

Table S1. Curated cytokines with annotation

Cytokine symbol	Range in healthy controls (mean ± SEM)	Description
**CD40 LG**	2571 ± 382.5	CD40LG-CD40 ligand; A cytokine that binds to CD40/TNFRSF5, co-stimulates T-cell proliferation, enhances the production of IL4 and IL10 and CD28 co-stimulation. It also induces the activation of NF-kappa-B, kinases MAPK8, PAK2 in T- cells and tyrosine phosphorylation of CD28. In addition, it facilitates B-cell proliferation, IgE production and contributes to immunoglobulin class switching.
**CSF2**	17.44 ± 1.310	Granulocyte-macrophage colony-stimulating factor; A cytokine that enhances the growth and differentiation of various lineages from hematopoietic precursor cells such as granulocytes, macrophages, eosinophils and erythrocytes.
**Granzyme B**	39.13 ± 8.971	Granzyme B; An enzyme involves in cell lysis in cell-mediated immune responses. It activates the cascade of caspases responsible for apoptosis execution. Cleaves caspase-3, -7, -9 and 10 to give rise to active enzymes mediating apoptosis.
**IFN-α**	6.933 ± 0.7322	IFNA2-Interferon alpha-2; Antiviral cytokine that produced by macrophages.
**IFN-γ**	63.95 ± 10.24	Interferon-gamma; Antiviral activity cytokine produced by lymphocytes. Also, it has other functions, including activation of macrophages, antiproliferative effects on transformed cells, and potentiating the antiviral and antitumor effects of the type I interferons.
**IL-10**	73.25 ± 8.655	Interleukin-10; A cytokine produced by activated macrophages and T-helper cells. The primary function is to inhibit the synthesis of several cytokines, including IFN-gamma, IL-2, IL-3, TNF and GM-CSF.
**IL-12**	25.51 ± 2.878	Interleukin 12; A cytokine that acts on T and natural killer cells. It is expressed by activated macrophages that serve as an essential inducer of Th1 cells development and is essential for sustaining a sufficient number of memory/effector Th1 cells to mediate long-term protection to an intracellular pathogen.
**IL-13**	62.41 ± 5.853	Interleukin-13; A cytokine that is involved in the inhibition of inflammatory cytokine production. It synergizes with IL2 to regulate interferon-gamma synthesis. Also, it acts as a positive regulator of IL31RA expression in macrophages.
**IL-15**	3.912 ± 0.4492	Interleukin-15; A cytokine with the ability to stimulate T- lymphocytes' proliferation through the interaction with IL-2R.
**IL-17A**	4.522 ± 0.3544	Interleukin-17A; A ligand for IL17RA and IL17RC. It's involved in inducing stromal cells to produce pro-inflammatory and hematopoietic cytokines.
**IL-1-α**	9.452 ± 0.3065	Interleukin-1 alpha; A cytokine produced by activated macrophages. It stimulates thymocyte proliferation, B-cell maturation/proliferation, and fibroblast growth factor activity. Also, IL-1 is involved in the inflammatory response and the release of prostaglandin and collagenase from synovial cells.
**IL-1β**	4.377 ± 1.056	Interleukin 1 beta; A member of the IL1 family produced by activated macrophages as a proprotein, which is proteolytically processed to its active form by caspase 1 (CASP1/ICE).<br><br>Mediator of the inflammatory response, cell proliferation, differentiation, and apoptosis.<br><br>IL-1B is involved in osteoarthritis pathogenesis. Recently, it has been found that Patients with severe COVID-19 infection showed high levels of IL-1B in bronchial alveolar lavage fluid samples.
**IL-1Ra**	694.7 ± 317.3	Interleukin-1 receptor type 1; binds to IL1A, IL1B and IL1RN receptors to form IL-1 receptor complex, which mediates the activation of NF-kappa-B, MAPK and other pathways. IL-1Ra is one of the antagonistic cytokines in the IL-1 superfamily.

**IL-2**	5.076 ± 0.5005	Interleukin-2; A cytokine produced by T-cells in response to antigenic or mitogenic stimulation. It's essential for T-cell proliferation and regulation of the immune response. IL-2 can stimulate B-cells, monocytes, lymphokine-activated killer cells, natural killer cells, and glioma cells.
**IL-33**	11.74 ± 0.9959	Interleukin-33; A cytokine that binds to and signals through the IL1RL1/ST2 receptor, which activates NF-kappa-B and MAPK signaling pathways in target cells. Involve in the maturation of Th2 cells inducing the secretion of T-helper type 2-associated cytokines. More, it's involved in the activation of mast cells, basophils, eosinophils and natural killer cells. Acts as a chemoattractant for Th2 cells and may function as an "alarmin" that amplifies immune responses during tissue injury.
**IL-4**	1.002 ± 0.07853	Interleukin-4; Involved in B-cell activation processes, expression of class II MHC molecules on resting B-cells and secretion and cell surface expression of IgE and IgG1. Other functions include regulating the expression of the low-affinity Fc receptor for IgE (CD23) on both lymphocytes and monocytes and IL31RA expression in macrophages.
**IL-6**	10.64 ± 1.627	Interleukin-6; A cytokine with an essential role in the differentiation of B-cells into Ig- secreting cells, monocyte differentiation and generation of T(H)17 cells. Acts on B-cells, T-cells, hepatocytes, hematopoietic progenitor cells, CNS cells and myokine. Acts to increase the breakdown of fats and to improve insulin resistance. However, it may induce myeloma and plas.
**CXCL8**	8.505 ± 1.751	CXCL8-Interleukin-8 is a chemotactic factor released from several cell types in response to an inflammatory stimulus to attract neutrophils, basophils, and T-cells. It is essential in neutrophil activation.
**CXCL10**	54.88 ± 5.384	C-X-C motif chemokine 10; A chemotactic for monocytes and T-lymphocytes. Binds to CXCR3; Belongs to the intracrine alpha (chemokine CxC) family.
**CCL2**	211.8 ± 18.31	C-C motif chemokine 2; A chemotactic factor that attracts monocytes and basophils. It has monocyte anti-tumor activity and was implicated in the pathogenesis of diseases characterized by monocytic infiltrates, like psoriasis, rheumatoid arthritis, or atherosclerosis.
**MIP1alpha**	20.21 ± 7.637	C-C chemokine receptor type 1; The ligand for this receptor is MIP-1-alpha, MIP-1-delta, RANTES, and MCP-3 and, less efficiently, to MIP-1- beta or MCP-1. Subsequently, it transduces a signal by increasing the intracellular calcium ions level, recruitment of effector immune cells to the site of inflammation and stem cell proliferation.
**MIP-1β**	157.2 ± 8.942	Chemokine 4; A monokine with inflammatory and chemokinetic properties typically binds to CCR5. It is a major HIV-suppressive factor produced by CD8+ T-cells. The processed form MIP-1-beta retains the ability to induce down-modulation of surface expression of the chemokine receptor CCR5 and to inhibit the CCR5- mediated entry of HIV-1 in T-cells.
**PD-L1**	131.9 ± 19.41	CD-274 - Programmed cell death 1 ligand 1; Involved in the co-stimulatory signal, essential for T-cell proliferation and production of IL10 and IFNG, in an IL2- dependent and a PDCD1-independent manner. Interaction with PDCD1 inhibits T-cell proliferation and cytokine production.
**TNF-α**	13.78 ± 1.471	Tumor necrosis factor-alpha; A proinflammatory cytokine that is mainly secreted by macrophages. It functions through the binding with its receptors TNFRSF1A/TNFR1 and TNFRSF1B/TNFBR. In addition, it involves the regulation of cell proliferation, differentiation, apoptosis, lipid metabolism, and coagulation.

Table S2: Multivariate analysis with Bonferroni multiple testing.

Table reports intra-groups comparisons identified to be significant. Bonferroni multiple group testing was only applicable to variables with 3 or more categorical groups.

Variable	Cytokine/ bio-chemical marker	Test	p-value (Sig.)
Severity	IL-1 alpha	Mild vs Moderate	0.005 (Mild > Moderate)
		Mild vs Severe	0.009 (Mild > Severe)
	IL-4	Mild vs Moderate	0.006 (Mild > Moderate)
		Mild vs Severe	0.012 (Mild > Severe)
	IL-10	Moderate vs Severe	0.001 (Severe > Moderate)
	IL-13	Mild vs Moderate	0.036 (Mild > Moderate)
	ANC	Moderate vs Severe	0.010 (Severe > Moderate)
	ALC	Moderate vs Severe	0.049 (Severe > Moderate)
	CRP	Moderate vs Severe	0.016 (Severe > Moderate)
	Ferritin	Moderate vs Severe	0.023 (Severe > Moderate)
	LDH	Moderate vs Severe	0.021 (Severe > Moderate)
	BUN	Moderate vs Severe	0.018 (Severe > Moderate)
	WBC	Moderate vs Severe	0.002 (Severe > Moderate)
Oxygen Support	IL-1 beta	None vs Mechanical ventilation	0.037 (None > Mechanical)
	IL-10	None vs Mechanical ventilation	0.011 (Mechanical > None)
		Non-invasive ventilation vs Mechanical ventilation	0.001 (Mechanical > Non-invasive)
	ANC	Non-invasive ventilation vs Mechanical ventilation	0.006 (Mechanical > Non-invasive)
	LDH	Non-invasive ventilation vs Mechanical ventilation	0.015 (Mechanical > Non-invasive)
	BUN	Non-invasive ventilation vs Mechanical ventilation	0.032 (Mechanical > Non-invasive)
	WBC	Non-invasive ventilation vs Mechanical ventilation	0.002 (Mechanical > Non-invasive)
Radiology	IFNG	None vs Consolidation	0.037 (None > Consolidation)
		None vs Ground-glass opacities	0.029 (None > Ground glass opacities)
	IL1Ra	None vs Pneumothorax	0.002 (Pneumothorax > None)
		Consolidation vs Pneumothorax	0.001 (Pneumothorax > Consolidation)
		Ground-glass opacities vs Pneumothorax	0.005 (Pneumothorax > Ground-glass opacities)
	IL6	None vs Pneumothorax	0.001 (Pneumothorax > None)
		Consolidation vs Pneumothorax	0.000 (Pneumothorax > Consolidation)
		Ground-glass opacities vs Pneumothorax	0.001 (Pneumothorax > Ground-glass opacities)
	MCP-1	None vs Pneumothorax	0.003 (Pneumothorax > None)
		Consolidation vs Pneumothorax	0.003 (Pneumothorax > Consolidation)
		Ground-glass opacities vs Pneumothorax	0.003 (Pneumothorax > Ground-glass opacities)
	PD-L1	None vs Consolidation	0.040 (None > Consolidation)
	D-Dimer	None vs Pneumothorax	0.002 (Pneumothorax > None)
		Consolidation vs Pneumothorax	0.001 (Pneumothorax > Consolidation)
		Ground-glass opacities vs Pneumothorax	0.000 (Pneumothorax > Ground-glass opacities)
Hb	PD-L1	Below normal vs Above normal	0.008 (above normal > below normal)
		Normal vs Above normal	0.007 (above normal > normal)
	Albumin	Below normal vs Above normal	0.024 (Above normal > Below normal)
BUN	IFNG	Below normal vs above normal	0.042 (Below normal > Above normal)
	IL1beta	Below normal vs normal	0.037 (below normal > normal)
		Below normal vs Above normal	0.013 (Below normal > Above normal)
	CRP	Normal vs above normal	0.030 (Above normal > Normal)
Creatinine	CD40LG	Below normal vs normal	0.024 (Below normal > Normal)
		Below normal vs Above normal	0.019 (Below normal > Above normal)
	IFNG	Below normal vs normal	0.012 (Below normal > Normal)
		Below normal vs Above normal	0.004 (Below normal > Above normal)
	IL1alpha	Below normal vs normal	0.002 (Below normal > Normal)
		Below normal vs Above normal	0.003 (Below normal > Above normal)
	IL4	Below normal vs normal	0.006 (Below normal > Normal)
		Below normal vs Above normal	0.014 (Below normal > Above normal)
	IL10	Normal vs above normal	0.004 (Below normal > Normal)
	IL12p70	Below normal vs normal	0.004 (Below normal > Normal
		Below normal vs Above normal	0.025 (Below normal > Above normal)
	IL13	Below normal vs normal	0.035 (Below normal > Normal)

	CRP	Normal vs Above Normal	0.001 (Above normal > Normal)
	D-Dimer	Normal vs Above Normal	0.029 (Above normal > Normal)
	BUN	Normal vs Above Normal	0.028 (Above normal > Normal)
	Platelets	Below normal vs Normal	0.033 (Below normal > Normal)
		Below normal vs Above Normal	0.004 (Below normal > Above normal)
ALC	IL13	Below normal vs above normal	0.023 (Above normal > Below Normal)
	ANC/ALC	Below normal vs Normal	0.002 (Below normal > Normal)
		Below normal vs Above normal	0.011 (Below normal > Above normal)

Table S3: ROC predictive efficacy analysis of each candidate marker identified using the two mathematical models (Regression and ANOVA).

List of markers that only showed a significant predictive capacity and area under the curve (AUC) value of ≥0.7. List of performance categories including area AUC, sensitivity, and specificity. Analysis is performed under the nonparametric assumption.

Variable (positive state)	Test Result Variable	AUC	Std. Error	Asymptotic Sig.	Asymptotic 95% Confidence Interval		Cutoff value (greater than or equal to)	Sensitivity	Specificity	PPV	NPV	PLR	NLR	positive	negative	accuracy
					Lower Bound	Upper Bound										
Severity (Severe)	IL10 (pg/mL)	0.83	0.07	0	0.7	0.97	204.5	0.82	0.8	0.777411377	0.890354493	4.1	0.225	0.4852	0.5148	0.8092
	PDL1 (pg/mL)	0.7	0.09	0.04	0.52	0.88	117.27	0.71	0.65	0.633436773	0.680760279	2.028571429	0.446153846	0.5156	0.4844	0.6776
	Ferritin (ng/mL)	0.78	0.08	0.01	0.62	0.93	724	0.75	0.75	0.70212766	0.893617021	3	0.333333333	0.47	0.505851064	0.75
	LDH (U/L)	0.76	0.09	0.01	0.59	0.93	325	0.79	0.65	0.610671192	0.723039216	2.257142857	0.323076923	0.5304	0.4696	0.7074
	WBC (10^3/µL)	0.76	0.09	0.01	0.59	0.93	10.25	0.77	0.65	0.652061856	0.646170839	2.2	0.353846154	0.5432	0.4568	0.7052
	BUN (mg/dL)	0.87	0.06	0	0.75	0.99	28.27	0.69	0.83	0.746138002	1.239443872	4.058823529	0.373493976	0.3884	0.6116	0.7712
Oxygen support (mechanical ventilation)	IL10 (pg/mL)	0.87	0.06	0	0.75	0.98	204.5	0.83	0.84	0.904100812	0.879111491	5.1875	0.202380952	0.4682	0.5318	0.8354
	WBC (10^3/µL)	0.78	0.08	0	0.61	0.94	10.25	0.78	0.68	0.748306998	0.626787058	2.4375	0.323529412	0.5316	0.4684	0.726
	BUN (mg/dL)	0.89	0.06	0	0.78	1	19.6	0.86	0.71	0.708142726	0.714547118	2.965517241	0.197183099	0.5465	0.4535	0.7775
	LDH (U/L)	0.79	0.08	0	0.63	0.95	325	0.8	0.68	0.662650602	0.71686747	2.5	0.294117647	0.5312	0.4688	0.7328
	Ferritin (ng/mL)	0.79	0.08	0	0.64	0.95	724	0.77	0.79	0.764792899	0.884826712	3.666666667	0.291139241	0.4732	0.5268	0.7806
	CRP (mg/L)	0.77	0.08	0.01	0.61	0.92	30.7	0.78	0.72	0.735849057	0.679245283	2.785714286	0.305555556	0.53	0.47	0.75

	ANC (10^3/µL)	0.74	0.11	0.02	0.53	0.96	7.35	0.75	0.63	0.564453879	0.741605558	2.027027027	0.396825397	0.5182	0.4818	0.6852
Radiology (pneumo-thorax)	IL1Ra (pg/mL)	0.98	0.02	0.02	0.94	1	22327.19	1	0.97	0.696807649	9.803219359	33.33333333	0	0.092565	0.907435	0.971935
	D-Dimer (µg/mL)	0.97	0.04	0.03	0.89	1	6.79	1	0.93	0.496211101	6.693195369	14.28571429	0	0.129985	0.870015	0.934515
	ALT (U/L)	0.99	0	0.02	1	1	203	0.99	0.98	0.773390662	11.10385757	49.5	0.010204082	0.082565	0.917435	0.980645
AST (above normal)	IL6 (pg/mL)	0.82	0.09	0.02	0.64	1	11.91	0.79	0.71	0.803389831	0.481355932	2.724137931	0.295774648	0.59	0.41	0.758
	CRP (mg/L)	0.82	0.1	0.02	0.63	1	11.5	0.69	0.71	0.815454545	0.451818182	2.379310345	0.436619718	0.55	0.45	0.697
	Creatinine (mg/dL)	0.83	0.09	0.02	0.66	1	0.8	0.86	0.57	0.80239521	0.261941234	2	0.245614035	0.7181	0.2819	0.7643
	LDH (U/L)	0.89	0.07	0	0.75	1	223.5	0.86	0.57	0.80239521	0.261941234	2	0.245614035	0.7181	0.2819	0.7643
Fatality (Deceased)	WBC (10^3/µL)	0.84	0.07	0.01	0.7	0.98	12.9	0.86	0.77	0.467257649	1.783528739	3.739130435	0.181818182	0.3497	0.6503	0.7871
	ANC (10^3/µL)	0.89	0.01	0.01	0.74	1	11.5	0.8	0.89	0.58076225	3.392014519	7.272727273	0.224719101	0.2204	0.7796	0.8756
	ANC/ALC (10^3/µL)	0.87	0.11	0.01	0.66	1	15.4	0.8	0.92	0.655737705	3.959016393	10	0.217391304	0.1952	0.8048	0.9008

R Code: RNAseq analysis:

```
# RNAseq analysis

library("DESeq2")
library("gplots")
library("RColorBrewer")

setwd("")


tabx <- read.table("input.txt", header=TRUE, sep="\t", quote="", comment.char="",
na.strings="#N/A")
bckCountTable <- tabx[,-1]
head(bckCountTable)

# condition <- factor(c("A","B","C","D"))

samples <- data.frame(row.names = colnames(bckCountTable), condition =
as.factor(c(rep("FI",4),rep("SI",4))))

dds <- DESeqDataSetFromMatrix(countData = bckCountTable, colData = samples,
design = ~condition)

deseqrna <- DESeq(dds)

plotDispEsts(deseqrna, ylim = c(1e-6, 1e1))



# Check assay using assay(rld)[1:3, 1:7]

plot(assay(deseqrna)[,c(1,2)], col="#00000020", pch=20, cex=0.3)

sampleDists <- dist(t(assay(deseqrna)))
as.matrix(sampleDists)[1:3, 1:3]


sampleDistMatrix <- as.matrix(sampleDists)
rownames(sampleDistMatrix) <- paste(deseqrna$condition, sep="-")
colnames(sampleDistMatrix) <- paste(deseqrna$condition, sep="-")

# Heatmap code

colours = colorRampPalette(rev(brewer.pal(9, "Blues")))(255)
heatmap.2(sampleDistMatrix, trace="none", col=colours)


topVarGenes <- head(order(rowVars(assay(deseqrna)), decreasing=TRUE), 2000)
heatmap.2(assay(deseqrna)[topVarGenes,], scale="row", trace="none",
dendrogram="column", col = colorRampPalette(rev(brewer.pal(9, "RdBu")))(255),
ColSideColors = c(A="gray", B="darkgreen",
C="orange")[colData(deseqrna)$condition])

resall = results(deseqrna)
```

```
# NGS QC code

plotMA(resall, ylim = c(-3, 3) )

hist(resall$pvalue, breaks=20, col="grey", ylim = c(0, 1500))

write.csv(resall,file="output.txt")
```

R code: clustering and heatmap in R proper with row labelling

```
# Unsupervised clustering

library(gplots)
library(RColorBrewer)

setwd("Path")

log2.ratio<-read.table("input", header= TRUE, sep="\t", quote="",
comment.char="", na.strings="#N/A")

log2.ratios <- log2.ratio[2:length(log2.ratio)]


heatmapcam <- function (x, Rowv = TRUE, Colv = if (symm) "Rowv" else TRUE,
distfun = dist, hclustfun = hclust, dendrogram = c("both", "row", "column",
"none"), symm = FALSE, scale = c("none", "row", "column"), na.rm = TRUE, revC =
identical(Colv, "Rowv"), add.expr, breaks, col = "heat.colors", colsep, rowsep,
sepcolor = "white", sepwidth = c(0.05, 0.05), cellnote, notecex = 1, notecol =
"cyan", na.color = par("bg"), trace = c("column", "row", "both", "none"),
tracecol = "cyan", hline = median(breaks), vline = median(breaks), linecol =
tracecol, margins = c(5,5), ColSideColors, RowSideColors, cexRow = 0.2 +
1/log10(nr), cexCol = 0.2 + 1/log10(nc), labRow = NULL, labCol = NULL, key =
TRUE, keysize = 1.5, density.info = c("histogram", "density", "none"), denscol =
tracecol, symkey = min(x < 0, na.rm = TRUE), densadj = 0.25, main = NULL, xlab =
NULL, ylab = NULL, ...)
{
    scale01 <- function(x, low = min(x), high = max(x))
    {
        x <- (x - low)/(high - low)
        x
    }
    scale <- if (symm && missing(scale))
        "none"
    else match.arg(scale)
    dendrogram <- match.arg(dendrogram)
    trace <- match.arg(trace)
    density.info <- match.arg(density.info)

    if (!missing(breaks) && (scale != "none"))
        warning("Using scale=\"row\" or scale=\"column\" when breaks are",
            "specified can produce unpredictable results.", "Please consider
using only one or the other.")
    if ((Colv == "Rowv") && (!isTRUE(Rowv) || is.null(Rowv)))
        Colv <- FALSE
    if (length(di <- dim(x)) != 2 || !is.numeric(x))
        stop("`x' must be a numeric matrix")
    nr <- di[1]
    nc <- di[2]
    if (nr <= 1 || nc <= 1)
        stop("`x' must have at least 2 rows and 2 columns")
    if (!is.numeric(margins) || length(margins) != 2)
        stop("`margins' must be a numeric vector of length 2")
    if (missing(cellnote))
        cellnote <- matrix("", ncol = ncol(x), nrow = nrow(x))
```

```
if (!inherits(Rowv, "dendrogram")) {
    if (((!isTRUE(Rowv)) || (is.null(Rowv))) && (dendrogram %in%
        c("both", "row"))) {
        if (is.logical(Colv) && (Colv))
            dendrogram <- "column"
        else dedrogram <- "none"
        warning("Discrepancy: Rowv is FALSE, while dendrogram is `",
            dendrogram, "'. Omitting row dendogram.")
    }
}
if (!inherits(Colv, "dendrogram")) {
    if (((!isTRUE(Colv)) || (is.null(Colv))) && (dendrogram %in%
        c("both", "column"))) {
        if (is.logical(Rowv) && (Rowv))
            dendrogram <- "row"
        else dendrogram <- "none"
        warning("Discrepancy: Colv is FALSE, while dendrogram is `",
            dendrogram, "'. Omitting column dendogram.")
    }
}
if (inherits(Rowv, "dendrogram")) {
    ddr <- Rowv
    rowInd <- order.dendrogram(ddr)
}
else if (is.integer(Rowv)) {
    hcr <- hclustfun(distfun(x))
    ddr <- as.dendrogram(hcr)
    ddr <- reorder(ddr, Rowv)
    rowInd <- order.dendrogram(ddr)
    if (nr != length(rowInd))
        stop("row dendrogram ordering gave index of wrong length")
}
else if (isTRUE(Rowv)) {
    Rowv <- rowMeans(x, na.rm = na.rm)
    hcr <- hclustfun(distfun(x))
    ddr <- as.dendrogram(hcr)
    ddr <- reorder(ddr, Rowv)
    rowInd <- order.dendrogram(ddr)
    if (nr != length(rowInd))
        stop("row dendrogram ordering gave index of wrong length")
}
else {
    rowInd <- nr:1
}
if (inherits(Colv, "dendrogram")) {
    ddc <- Colv
    colInd <- order.dendrogram(ddc)
}
else if (identical(Colv, "Rowv")) {
    if (nr != nc)
        stop("Colv = \"Rowv\" but nrow(x) != ncol(x)")
    if (exists("ddr")) {
        ddc <- ddr
        colInd <- order.dendrogram(ddc)
```

```
    }
    else colInd <- rowInd
}
else if (is.integer(Colv)) {
    hcc <- hclustfun(distfun(if (symm)
        x
    else t(x)))
    ddc <- as.dendrogram(hcc)
    ddc <- reorder(ddc, Colv)
    colInd <- order.dendrogram(ddc)
    if (nc != length(colInd))
        stop("column dendrogram ordering gave index of wrong length")
}
else if (isTRUE(Colv)) {
    Colv <- colMeans(x, na.rm = na.rm)
    hcc <- hclustfun(distfun(if (symm)
        x
    else t(x)))
    ddc <- as.dendrogram(hcc)
    ddc <- reorder(ddc, Colv)
    colInd <- order.dendrogram(ddc)
    if (nc != length(colInd))
        stop("column dendrogram ordering gave index of wrong length")
}
else {
    colInd <- 1:nc
}
x <- x[rowInd, colInd]
x.unscaled <- x
cellnote <- cellnote[rowInd, colInd]
if (is.null(labRow))
    labRow <- if (is.null(rownames(x)))
        (1:nr)[rowInd]
    else rownames(x)
else labRow <- labRow[rowInd]
if (is.null(labCol))
    labCol <- if (is.null(colnames(x)))
        (1:nc)[colInd]
    else colnames(x)
else labCol <- labCol[colInd]
if (scale == "row") {
    x <- sweep(x, 1, rowMeans(x, na.rm = na.rm))
    sx <- apply(x, 1, sd, na.rm = na.rm)
    x <- sweep(x, 1, sx, "/")
}
else if (scale == "column") {
    x <- sweep(x, 2, colMeans(x, na.rm = na.rm))
    sx <- apply(x, 2, sd, na.rm = na.rm)
    x <- sweep(x, 2, sx, "/")
}
if (missing(breaks) || is.null(breaks) || length(breaks) < 1)
    if (missing(col))
        breaks <- 16
    else breaks <- length(col) + 1
```

```
    if (length(breaks) == 1) {
        breaks <- seq(min(x, na.rm = na.rm), max(x, na.rm = na.rm),
            length = breaks)
    }


print(breaks)
cat("length breaks = \n", length(breaks))
cat("\n")

    nbr <- length(breaks)
    ncol <- length(breaks) - 1
    if (class(col) == "function")
        col <- col(ncol)
    else if (is.character(col) && length(col) == 1)
        col <- do.call(col, list(ncol))
    min.breaks <- min(breaks)
    max.breaks <- max(breaks)
    x[] <- ifelse(x < min.breaks, min.breaks, x)
    x[] <- ifelse(x > max.breaks, max.breaks, x)
    lmat <- rbind(4:3, 2:1)
    lhei <- lwid <- c(keysize, 4)
    if (!missing(ColSideColors)) {
        if (!is.character(ColSideColors) || length(ColSideColors) !=
            nc)
            stop("'ColSideColors' must be a character vector of length ncol(x)")
        lmat <- rbind(lmat[1, ] + 1, c(NA, 1), lmat[2, ] + 1)
        lhei <- c(lhei[1], 0.2, lhei[2])
    }
    if (!missing(RowSideColors)) {
        if (!is.character(RowSideColors) || length(RowSideColors) !=
            nr)
            stop("'RowSideColors' must be a character vector of length nrow(x)")
        lmat <- cbind(lmat[, 1] + 1, c(rep(NA, nrow(lmat) - 1),
            1), lmat[, 2] + 1)
        lwid <- c(lwid[1], 0.2, lwid[2])
    }
    lmat[is.na(lmat)] <- 0
    op <- par(no.readonly = TRUE)
    on.exit(par(op))
    layout(lmat, widths = lwid, heights = lhei, respect = FALSE)
    if (!missing(RowSideColors)) {
        par(mar = c(margins[1], 0, 0, 0.5))
        image(rbind(1:nr), col = RowSideColors[rowInd], axes = FALSE)
    }
    if (!missing(ColSideColors)) {
        par(mar = c(0.5, 0, 0, margins[2]))
        image(cbind(1:nc), col = ColSideColors[colInd], axes = FALSE)
    }
    par(mar = c(margins[1], 0, 0, margins[2]))
    if (!symm || scale != "none") {
        x <- t(x)
        cellnote <- t(cellnote)
    }
```

```
if (revC) {
    iy <- nr:1
    ddr <- rev(ddr)
    x <- x[, iy]
    cellnote <- cellnote[, iy]
}
else iy <- 1:nr
image(1:nc, 1:nr, x, xlim = 0.5 + c(0, nc), ylim = 0.5 +
    c(0, nr), axes = FALSE, xlab = "", ylab = "", col = col,
    breaks = breaks, ...)
if (!invalid(na.color) & any(is.na(x))) {
    mmat <- ifelse(is.na(x), 1, NA)
    image(1:nc, 1:nr, mmat, axes = FALSE, xlab = "", ylab = "",
        col = na.color, add = TRUE)
}
axis(1, 1:nc, labels = labCol, las = 2, line = -0.5, tick = 0,
    cex.axis = cexCol)
if (!is.null(xlab))
    mtext(xlab, side = 1, line = margins[1] - 1.25)
axis(4, iy, labels = labRow, las = 2, line = -0.5, tick = 0,
    cex.axis = cexRow)
if (!is.null(ylab))
    mtext(ylab, side = 4, line = margins[2] - 1.25)
if (!missing(add.expr))
    eval(substitute(add.expr))
if (!missing(colsep))
    for (csep in colsep) rect(xleft = csep + 0.5, ybottom = rep(0,
        length(csep)), xright = csep + 0.5 + sepwidth[1],
        ytop = rep(ncol(x) + 1, csep), lty = 1, lwd = 1,
        col = sepcolor, border = sepcolor)
if (!missing(rowsep))
    for (rsep in rowsep) rect(xleft = 0, ybottom = (ncol(x) +
        1 - rsep) - 0.5, xright = ncol(x) + 1, ytop = (ncol(x) +
        1 - rsep) - 0.5 - sepwidth[2], lty = 1, lwd = 1,
        col = sepcolor, border = sepcolor)
min.scale <- min(breaks)
max.scale <- max(breaks)
x.scaled <- scale01(t(x), min.scale, max.scale)
if (trace %in% c("both", "column")) {
    for (i in colInd) {
        if (!is.null(vline)) {
            vline.vals <- scale01(vline, min.scale, max.scale)
            abline(v = i - 0.5 + vline.vals, col = linecol,
              lty = 2)
        }
        xv <- rep(i, nrow(x.scaled)) + x.scaled[, i] - 0.5
        xv <- c(xv[1], xv)
        yv <- 1:length(xv) - 0.5
        lines(x = xv, y = yv, lwd = 1, col = tracecol, type = "s")
    }
}
if (trace %in% c("both", "row")) {
    for (i in rowInd) {
        if (!is.null(hline)) {
```

```
            hline.vals <- scale01(hline, min.scale, max.scale)
            abline(h = i + hline, col = linecol, lty = 2)
        }
        yv <- rep(i, ncol(x.scaled)) + x.scaled[i, ] - 0.5
        yv <- rev(c(yv[1], yv))
        xv <- length(yv):1 - 0.5
        lines(x = xv, y = yv, lwd = 1, col = tracecol, type = "s")
    }
}
if (!missing(cellnote))
    text(x = c(row(cellnote)), y = c(col(cellnote)), labels = c(cellnote),
        col = notecol, cex = notecex)
par(mar = c(margins[1], 0, 0, 0))
if (dendrogram %in% c("both", "row")) {
    plot(ddr, horiz = TRUE, axes = FALSE, yaxs = "i", leaflab = "none")
}
else plot.new()
par(mar = c(0, 0, if (!is.null(main)) 5 else 0, margins[2]))
if (dendrogram %in% c("both", "column")) {
    plot(ddc, axes = FALSE, xaxs = "i", leaflab = "none")
}
else plot.new()
if (!is.null(main))
    title(main, cex.main = 1.5 * op[["cex.main"]])
if (key) {
    par(mar = c(5, 4, 2, 1), cex = 0.75)
    if (symkey) {
        max.raw <- max(abs(x), na.rm = TRUE)
        min.raw <- -max.raw
    }
    else {
        min.raw <- min(x, na.rm = TRUE)
        max.raw <- max(x, na.rm = TRUE)
    }
    z <- seq(min.raw, max.raw, length = length(col))
    image(z = matrix(z, ncol = 1), col = col, breaks = breaks,
        xaxt = "n", yaxt = "n")
    par(usr = c(0, 1, 0, 1))
    lv <- pretty(breaks)
    xv <- scale01(as.numeric(lv), min.raw, max.raw)
    axis(1, at = xv, labels = lv)
    if (scale == "row")
        mtext(side = 1, "Row Z-Score", line = 2)
    else if (scale == "column")
        mtext(side = 1, "Column Z-Score", line = 2)
    else mtext(side = 1, "Value", line = 2)
    if (density.info == "density") {
        dens <- density(x, adjust = densadj, na.rm = TRUE)
        omit <- dens$x < min(breaks) | dens$x > max(breaks)
        dens$x <- dens$x[-omit]
        dens$y <- dens$y[-omit]
        dens$x <- scale01(dens$x, min.raw, max.raw)
        lines(dens$x, dens$y/max(dens$y) * 0.95, col = denscol,
            lwd = 1)
```

```
            axis(2, at = pretty(dens$y)/max(dens$y) * 0.95, pretty(dens$y))
            title("Color Key\nand Density Plot")
            par(cex = 0.5)
            mtext(side = 2, "Density", line = 2)
        }
        else if (density.info == "histogram") {
            h <- hist(x, plot = FALSE, breaks = breaks)
            hx <- scale01(breaks, min.raw, max.raw)
            hy <- c(h$counts, h$counts[length(h$counts)])
            lines(hx, hy/max(hy) * 0.95, lwd = 1, type = "s",
                col = denscol)
            axis(2, at = pretty(hy)/max(hy) * 0.95, pretty(hy))
            title("Color Key\nand Histogram")
            par(cex = 0.5)
            mtext(side = 2, "Count", line = 2)
        }
        else title("Color Key")
    }
    else plot.new()
    invisible(list(rowInd = rowInd, colInd = colInd))
}



# "Threshold" the NA values to some arbitrary "large" distance.
# I wrote the following dist function computes distances and then replaces any NA
values with an arbitrarily
# large distance (10% greater then the largest actually distance).
# This function may be helpful for input into hclust because NA values are
replaced

na.dist <- function(x,...)
{
 t.dist <- dist(x,...)
 t.dist <- as.matrix(t.dist)
 t.limit <- 1.1*max(t.dist,na.rm=T)
 t.dist[is.na(t.dist)] <- t.limit
 t.dist <- as.dist(t.dist)
 return(t.dist)
}

ndist<-na.dist(t(log2.ratios), method="euclidean") # t = transpose
ntree<-hclust(ndist, method="ward.D2")
plot(ntree)


#Uses 256 shades of Red and Blue
heatcol<-colorRampPalette(brewer.pal(11, "RdBu"))(256)

csc <- heatcol[seq(from=1,to=256,length=109)]

#heatcol<-colorRampPalette(brewer.pal(nrow(mat), "RdBu"))(256)
```

```
row_param <- as.character(log2.ratio[,1])

rownames(log2.ratios) <- row_param

mat=data.matrix(log2.ratios)


x<-mat

dist.x <- dist(x,method="euclidean",diag=TRUE)
clust.x <- hclust(dist.x, method="ward.D2")
dist.y <- dist(t(x), method="euclidean",diag=TRUE)
clust.y <- hclust(dist.y,method="ward.D2")

# heatmapcam(x, Rowv=as.dendrogram(clust.x), Colv=as.dendrogram(clust.y),
symkey=FALSE, density.info="none", trace="none", scale="row", cexRow=0.5,
cexCol=0.5, col=heatcol, ColSideColors=csc)

# heatmapcam(x, Rowv=as.dendrogram(clust.x), Colv=as.dendrogram(clust.y),
symkey=FALSE, density.info="none", trace="none", cexRow=0.5, cexCol=0.5, breaks =
26, col = greenred(25))

heatmapcam(x, Rowv=as.dendrogram(clust.x), Colv=as.dendrogram(clust.y),
symkey=FALSE, density.info="none", trace="none", cexRow=0.5, cexCol=0.5, col =
colorpanel(n=99,low="green",mid="black",high="red"),
breaks=c(seq(0,300,length.out=50),seq(300,1000,length.out=50)))


col<- colorRampPalette(c("red", "white", "blue"))(256)
col <- colorRampPalette(brewer.pal(10, "RdYlBu"))(256)

row.names(x) <- log2.ratio[,1]


col<- colorRampPalette(c("red", "green", "black"))(256)

heatmap.2(x, scale = "row", col = greenred(100), trace = "none", cexRow=0.8,
cexCol=0.8, density.info = "none")
```
